# Role of Chemokines in the Biology of Cholangiocarcinoma

**DOI:** 10.3390/cancers12082215

**Published:** 2020-08-07

**Authors:** Alessandra Caligiuri, Mirella Pastore, Giulia Lori, Chiara Raggi, Giovanni Di Maira, Fabio Marra, Alessandra Gentilini

**Affiliations:** Department of Experimental and Clinical Medicine, University of Florence, Largo Brambilla 3, 50134 Florence, Italy; alessandra.caligiuri@unifi.it (A.C.); mirella.pastore@unifi.it (M.P.); giulia.lori@unifi.it (G.L.); chiara.raggi@unifi.it (C.R.); giovanni.dimaira@unifi.it (G.D.M.)

**Keywords:** cholangiocarcinoma, chemokines, cancer associated fibroblasts

## Abstract

Cholangiocarcinoma (CCA), a heterogeneous tumor with poor prognosis, can arise at any level in the biliary tree. It may derive from epithelial cells in the biliary tracts and peribiliary glands and possibly from progenitor cells or even hepatocytes. Several risk factors are responsible for CCA onset, however an inflammatory milieu nearby the biliary tree represents the most common condition favoring CCA development. Chemokines play a key role in driving the immunological response upon liver injury and may sustain tumor initiation and development. Chemokine receptor-dependent pathways influence the interplay among various cellular components, resulting in remodeling of the hepatic microenvironment towards a pro-inflammatory, pro-fibrogenic, pro-angiogenic and pre-neoplastic setting. Moreover, once tumor develops, chemokine signaling may influence its progression. Here we review the role of chemokines in the regulation of CCA development and progression, and the modulation of angiogenesis, metastasis and immune control. The potential role of chemokines and their receptors as possible biomarkers and/or therapeutic targets for hepatobiliary cancer is also discussed.

## 1. Introduction

Cholangiocarcinoma (CCA) comprises a heterogeneous group of biliary cancers, which can originate from cholangiocytes located at any portion of biliary tree [1]. Based on the anatomical position, this tumor can be classified in intrahepatic (iCCA) and extrahepatic (eCCA) CCA, this latter further divided into perihilar (pCCA) and distal CCA (dCCA), depending on the site within the biliary system [1,2].

CCA represents the second most frequent hepatic malignancy, accounting for 10–20% of all primary liver cancers [2,3] and its incidence is increasing dramatically [3]; accordingly, CCA mortality has increased worldwide in the last decades [4,5,6,7,8].

CCA is commonly asymptomatic at early stages and is often diagnosed when the disease is disseminated [9,10]. This limits the effectiveness of the current therapeutic strategies, which are preferably based on surgical resection, because antitumor drugs have only limited effects, in part owing to the high chemoresistance of this tumor [9,10]. As a result, CCA prognosis is dismal, with a 5-year survival lower than 20% [9,10].

Although the resistance to drugs is an intrinsic feature of malignant cholangiocytes [11], an additional role is played by the extensive desmoplastic microenvironment wherein neoplastic cells are embedded. Recent data support the concept that the desmoplastic stroma, that is a main feature of CCA, contributes to the decreased sensitivity of this tumor to drug-cytotoxicity, hampering responses to chemotherapy and resulting in a poor clinical outcome [1,2,4].

Although most CCAs are diagnosed de novo without an apparent liver disease background, there are well-established risk factors indicating that the appearance of CCA is favored in the context of chronic inflammatory conditions of the biliary tree (such as primary sclerosing cholangitis (PSC)) [1]. In this setting several autocrine, paracrine and endocrine signals concur to modify the environment in which tumors eventually develop: a spectrum of soluble factors (growth factors, cytokines, chemokines and proteases), released in a dysregulated fashion, sustain the inflammatory response and induce the ECM remodeling, promoting CCA initiation and progress [12]. Among these, chemokines are emerging as key factors in the complex network of events involved in development, invasiveness and immune evasion of several malignancies, including CCA.

In this review we will summarize the most recent findings on the role played by chemokine-induced signals in driving CCA malignancy. We will also focus on the possible mechanisms responsible for CCA drug resistance involving chemokine systems. Finally, we will highlight recent developments on the role of chemokines and their receptors as possible predictive and prognostic biomarkers and their potential employment as therapeutic targets for hepatobiliary cancers, also discussing the current limitations of this approach.

## 2. CCA and CCA-Associated Tumor Microenvironment

Stromal desmoplasia is a prominent histopathological hallmark of CCA that profoundly affects neoplastic ducts, contributing to CCA pathogenesis [5,6].

The highly reactive microenvironment is a dynamic and sophisticated compartment consisting of activated fibroblasts (cancer-associated fibroblasts, (CAFs)), endothelial and immune cells (tumor-associated macrophages (TAMs), neutrophils, natural killer (NK) cells, T and B lymphocytes) embedded in a non-physiological, fibrillar ECM [1].

CAFs, the major cellular components of desmoplastic stroma, play a critical part in biliary carcinogenesis, from neoplastic transformation to tumor dissemination. Their activation is due to a wide range of soluble mediators produced by tumor cells, as well as by the multiple inflammatory cells populating the desmoplastic stroma. By secreting growth factors, cytokines and chemokines (CXCL2, CXCL12, CXCL14), CAFs recruit inflammatory and endothelial cells, sustaining neoangiogenesis and lymphangiogenesis [7]. Moreover, CAFs elicit ECM structural changes, further supporting desmoplastic stroma and promoting cancer invasiveness [7].

Among the immune cell types infiltrating the desmoplastic stroma, TAMs play a crucial role in regulating angiogenesis, lymphangiogenesis, tumor proliferation and modulating ECM changes [1], through the release of inflammatory mediators [8].

Tumor-infiltrating lymphocytes (TILs) represent a highly heterogeneous populations [9,10] that comprise CD8+ cytotoxic T cells, CD4+ T helper cells, Tregs and B lymphocytes. Whereas high levels of CD4+ and CD8+ within CCA microenvironment have been associated with better prognosis [10,11,12], low numbers of CD8+ TILs are correlated with poor overall survival [13]. Regarding B cells, no data on their pathogenic role of in CCA are available, even if high densities of CD20+ B cells have been observed in low-grade tumors and associated with a favorable overall survival [9,10]. Little is known regarding the pathogenic role of NK cells in CCA, although, according to recent studies, these cells seem to inhibit CCA growth and reduce tumor chemoresistance [11,12].

The role of neutrophils in CCA is still indefinite, even a significant commitment of infiltrated tumor-associated neutrophils (TANs) in CCA tissues has been reported [14].

**Figure 1:**
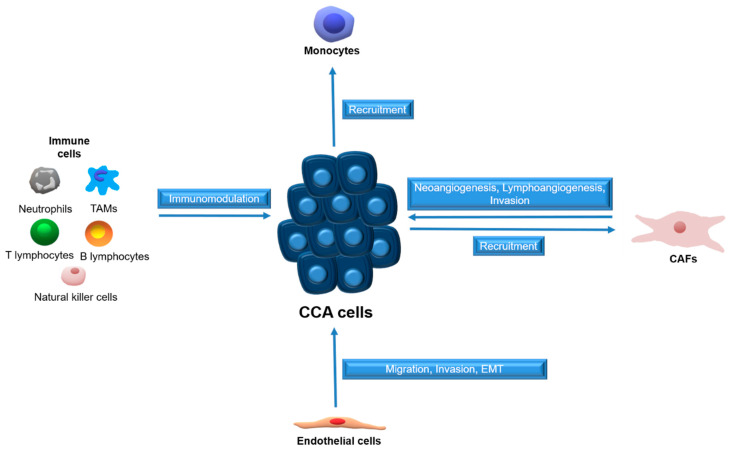
Schematic representation of paracrine effects between stromal cells and CCA cells. Tumor associated macrophages (TAMs), cancer-associated fibroblasts (CAFs), cholangiocarcinoma (CCA).

Tumors employ several mechanisms to establish a functional vascular system comprised of both blood and lymphatic vessels, to sustain cell growth [15,16]. CCA cells promote neo-vascularization by enhanced expression of angiogenic growth factors, whereas endothelial cells can release inflammatory chemokines to attract leukocytes and establish a pro-fibrotic and pro-angiogenic milieu, which in turn support migration, invasion and EMT [17]. The paracrine effects between CCA cells and surrounding stromal cells are summarized in Figure 1.

## 3. Chemokines Ligands and Receptors

Chemokines are a family of highly conserved small (8–12 kD) proteins, sharing the ability to chemoattract leukocytes. In humans, 48 chemokines have been identified, classified in four groups, according to the position of the first cysteine residues in their N-terminal sequence: XCL, CCL, CXCL and CX3CL, where X represents any other amino acid [18,19]. Chemokine signaling is transduced by G protein-coupled receptors, also divided in four groups (XCR, CCR, CXCR and CX3CR). Among the 19 receptors identified, most can bind to different chemokines, generally belonging to the same subfamily, with variable affinity and different functions [20]. Similarly, some chemokines bind and activate more than one receptor. As an important consequence of this promiscuity, chemokine-receptor interaction and the resulting signaling cascade are finely modulated, in concert with the modifications of the microenvironment.

Chemokines can also bind and activate a different category of receptors, named atypical receptors (ACKR1-6) [21], which show extreme ligand promiscuity. Although their functions are not fully elucidated, most of them appear to act as decoy receptors, negatively modulating the activation of “main” chemokine receptors [22]. ACKRs lack a G protein activation motif and ligand-receptor interaction leads to β-arrestin recruitment and subsequent internalization and degradation/recycling of the ligand-receptor complex, thus serving as a chemokine reservoir or scavenger [21,23,24]. Deregulation of ACKR expression has been reported in many tumors and appears to correlate with the metastatic process [25].

Chemokines were firstly reported as key effectors of immune and inflammatory reactions, driving recruitment and homing of leukocytes into infected or injured tissues [26]. Subsequently, an essential role in several pathophysiological processes, including organ development, tissue homeostasis, angiogenesis and cancer has been recognized [27]. According to their biological functions, chemokines can be distinguished in homeostatic, which are constitutively expressed in specific cell types and contribute to immune homeostasis, and inducible, whose expression is related to certain conditions, such as inflammatory responses. Concomitantly, leukocytes express a broad spectrum of receptors, making them susceptible to many chemokine ligands [28]. In particular, CC ligands mainly act on monocytes/macrophages and T cells, while CXCL1–8 primarily exert their chemotactic action on neutrophils and CXCL9-11 on T-cells. Finally, a few chemokines display both homeostatic and pro-inflammatory functions, depending on the localization and the timing of expression [28,29].

## 4. Regulation of Chemokine Expression and Effects

A variety of mechanisms have developed to control chemokine expression and/or activity, in order to ensure a proper cell trafficking and homing during innate and adaptive immune response. A number of polymorphisms have been identified in genes encoding chemokine and chemokine receptors, resulting in an altered expression/stability or improper ligand-receptor interaction. For instance, nine SNPs have been described in *CCR2* gene, associated with various disorders, including cancer [22]. Alternative splicing of precursor mRNAs has been observed for chemokine receptors or their ligands. Splice variants can exhibit different functions and be implicated in pathological conditions, such as cancer. Interaction with glycosaminoglycans (GAGs) is essential to maintain high chemokine levels in the site of release and altered chemokine binding to GAGs can result in impaired leukocytes extravasation [30]. GAG-chemokine interactions also influence the chemokine pattern and, consequently, the leukocyte populations recruited in specific areas. Finally, GAGs promote chemokine oligomerization preserving them by proteolytic cleavage and modulating chemokine-receptor linking [31]. Proteolytic cleavage can occur at either N- or C-terminal region by several proteases [32], including metalloproteases [33], dipeptidyl peptidase 4 (DPP4) [34] or cathepsin B [35]. Cleaved chemokines can display either reduced or increased activity, or different receptor selectivity. Inactivation of chemokines through proteolytic cleavage may be an efficient mechanism adopted by cancer cells to evade immune response [36], as recently demonstrated for CXCL9-11 and CX3CL1 [37]. Other post-translational modifications of chemokines and their receptors include O- and N-glycosylation, citrullination, ubiquitination, sulfation, nitration and nitrosylation [22]. These processes have been shown to affect protein localization, stability and clearance, as well as their chemotactic properties [22].

## 5. Chemokines and Cancer

Aberrant expression of chemokine ligands and receptors has been observed in several tumors, concurring to altered chemokine functions that contribute to tumorigenesis, sustained by inactivation of tumor suppressor genes, constitutive activation of transcription factors or deregulation of oncogenes regulating chemokines [38]. Indeed, in many tumors a constitutive activation of nuclear factor-κB (NF-κB) is associated with expression of chemokines that promote carcinogenesis [39]. Hypoxic conditions frequently occurring in tumor microenvironment lead to overexpression of chemokine ligands and receptors, both in cancer and stromal cells [40,41]. Cancer metabolism represents an additional element in chemokine regulation. Aerobic glycolysis and lactic acid were reported to induce NF-κB activity, and to increase CXCL8 expression and angiogenesis in breast and colon cancer [42]; ROS release has been associated with overexpression of CXCL14 and enhanced invasion and motility [43].

**Figure 2:**
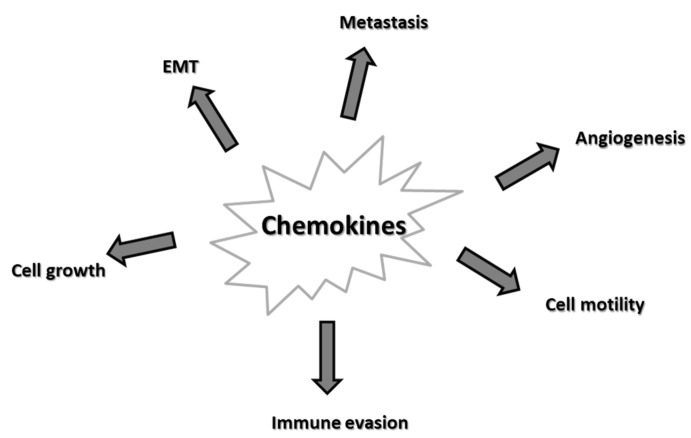
Major effects of chemokines on cancer. Epithelial-mesenchymal transition (EMT).

Alterations in the chemokine system are implicated in many aspects of tumorigenesis, as depicted in Figure 2 and listed below.

### 5.1. Tumor Growth

Most chemokine/GPCRs sustain cancer cell survival and proliferation, which are mainly mediated by activation of mitogen-activated protein kinases (MAPKs) [44,45] and PI3K/Akt [46] pathways. In contrast, some chemokine systems can transduce inhibitory signals, e.g., CCR1 [47] or CCR5 [48].

### 5.2. Epithelial-Mesenchymal Transition (EMT)

The CXCL8/CXCR1 system has been frequently associated to EMT [49,50,51]. Among atypical receptors, CXCR7 (ACKR3) has been reported to induce EMT and sustain tumor development in bladder cancer [46].

### 5.3. Angiogenesis

A high variety of chemokines directly or indirectly affect angiogenesis, with positive or negative actions. Angiogenic effects have been reported for CXCL1–3, CXCL5–6, CXCL8, CXCL12, CCL2, CCL11 and CCL16 [52,53]. In general, chemokines displaying the ELR motif, which allows leukocytes to roll on activated endothelium and migrate to the site of injury, are angiogenic [54]. Angiogenesis can be mediated by the expression of pro-angiogenic factors (such as vascular endothelial growth factor (VEGF), platelet derived growth factor (PDGF) and others), or directly promoting endothelial cell recruitment and proliferation. Alternatively, these chemokines can recruit immune cells, as neutrophils, dendritic cells (DCs), myeloid derived suppressor cells (MDSCs) and TAMs [55,56,57] able to secrete angiogenic factors [39,58,59]. MDSCs and TAMs can even adopt endothelial cell features, contributing to vessel formation [60].

### 5.4. Metastasis

Changes induced by chemokines and their receptors on endothelium are crucial for cancer cell migration, invasion and metastasis. Chemokines released by the tumor microenvironment (TME) increase vessel permeability, promoting intra/extravasation and migration of malignant cells expressing the appropriate receptors and driving them to distant organs [61]. A primary role is played by TAMs, whose recruitment/activation is mainly mediated by CCL2, although other proteins, as CCL3, CCL5, CCL8 [62] or CCL18 can be also effective. Some of these molecules, e.g., CCL3, CCL8, CCL22, further sustain chemokine secretion, thus favoring the accumulation of pro-metastatic immune cells [63].

### 5.5. Immune Evasion

As mentioned above, many tumors express proteinases able to process and inactivate chemokines, thus impairing leukocyte recruitment and host defense [37]. Cancer cells, as well as the diverse cell types of the surrounding stroma, produce cytokines and chemokines, such as CXCL5 and CXCL8 that induce neutrophil recruitment and phenotypical transition into pro-tumorigenic MDSCs [64,65,66]. In the TME, MDSCs exert pro-cancer actions secreting soluble factors able to suppress TIL trafficking and anti-cancer activity [67,68].

## 6. Chemokines and CCA

Molecular mechanisms favoring the development of a tumor reactive stroma (TRS) are crucial in the progression of CCA. Gene expression profiling of human CCA tissues identified a number of stromal-specific dysregulated genes correlated with poor clinical outcome, including genes encoding chemokines or chemokine receptors (CXCR4, CCR7, CCL2, CCL19, CCL21) [69]. CAFs have been identified as major contributors of soluble mediators with pro-tumorigenic functions, and CCA cells co-cultured with CAFs or exposed to CAF conditioned medium exhibit increased survival, proliferation and motility [70,71,72]. In addition, immunodeficient mice co-inoculated with CCA cells and myofibroblastic hepatic stellate cells (HSCs) showed higher tumor development respect to animals only injected with CCA cells [73,74]. Moreover, CAF depletion in TRS reduced tumor growth in a rat model of CCA [75]. Thus, chemokines involved in the cross-talk between tumor and stroma can modulate the biological activities of cancer cells, as growth and invasiveness, acting in autocrine or paracrine fashion [64]. Soluble factors secreted by CAFs also recruit and activate inflammatory and endothelial cells, providing additional mechanisms to sustain tumor progression and metastasis [76].

In addition, chemotactic factors released by both tumor and stromal cells contribute to recruitment and activation of TAMs [77]. Soluble mediators also induce the switch toward the M2 macrophage phenotype, although M1 and M2 features can often coexist [78]. Recently, Raggi et al. [19] have shown that CCA stem-like cells (CSC) can be involved in recruitment of circulating monocytes and their differentiation into TAMs. These CSC-associated TAMs co-express M1- (CXCL9 and CXCL10) and M2-related (CCL17 and CCL18) chemokines, suggesting that diverse TAM subpopulations can coexist in the TRS, displaying different phenotype and functions, depending on the components of the stromal milieu.

Under the chemotactic action of tumor-secreted chemokines, mesenchymal stem cells (MSCs) can be also recruited into the primary tumors. MSCs release soluble mediators that contribute to cancer progression, by promoting angiogenesis, impairing immune cell activity and increasing cancer invasiveness [79,80,81].

In the next sections, an in depth analysis of the main chemokine systems involved in biliary malignancies is presented. They are also summarized in Table 1.

## 7. CXCL12/CXCR4 and CXCL12/CXCR7

CXCL12, also known as stromal cell-derived factor-1 (SDF-1), is a member of the CXC chemokine subfamily expressed in many tissues and cell type [98]. CXCL12 binds to two different receptors: CXCR4 and CXCR7 [99]. In solid tumors, CXCL12 can be secreted both by cancer cells and CAFs [99], playing a role in paracrine or autocrine fashion, through either CXCR4 or CXCR7. CXCR4 is a highly conserved receptor expressed on various cell types, including several cancer cells [100]. In the circulatory system CXCR4 regulates hematopoietic stem cell homing to the bone marrow, and trafficking of hematopoietic cells and lymphocytes [101].

CXCR4/CXCL12 interaction triggers different downstream pathways in cancer cells or in several cells of TME, resulting in diverse biological responses, as angiogenesis, metastasis, proliferation and survival [102,103].

Upregulation of CXCR4 expression has been observed in different human cancers [104], and the CXCL12/CXCR4 axis is often considered a hallmark of cancer aggressiveness and correlates with tumor size, grade and recurrence [105,106,107,108], poor prognosis [106] and low survival [109]. In some tumors, it is also critical for drug-resistance [110,111].

CXCR7, also known as ACKR3 and previously considered only a scavenger receptor, binds to CXCL12 with higher affinity than CXCR4 [112]. Binding of CXCL12 to CXCR7 induces non-G protein-mediated β-arrestin accumulation and subsequent ERK activation [113].

CXCR7 and CXCR4 can form heterodimers, shifting the CXCL12-induced signaling from G-proteins-dependent to β-arrestin-dependent signals [114,115].

CXCR7 expression has been observed in fetal liver cells, activated endothelial cells and various tumor cells, as well as in malignancy associated blood vessels, where CXCR7 induces cell growth, survival and increased adhesion [116].

Increased expression of CXCR7 has been observed in pancreatic, prostate, liver, ovarian, kidney, colon, breast and lung cancer [116], and in general there is a positive correlation between CXCR7 expression and tumor malignancy [117]. In addition, high expression of CXCR7 confers a high risk of developing lymph node metastasis [118].

The role of CXCL12/CXCR4 axis in CCA has been extensively examined, mostly highlighting a paracrine function of this pathway. Ohira S. et al. [119] demonstrated that migration, but not proliferation, of iCCA cells is induced by CXCL12 released by WI-38 fibroblasts. Moreover, CXCR4 expression in cancer cells increased upon treatment with tumor necrosis factor α (TNF-α), released both by TAMs and tumor cells. In human iCCA tissues TNF-α is mainly expressed in infiltrating macrophages, while CXCR4 is present in cancer cells but not in non-neoplastic ducts and CXCL12 in stromal fibroblasts and, to a lesser extent, in tumor cells [119]. In another study, CXCL12 release by fibroblasts was downregulated by transforming growth factor-β (TGF-β) secreted by iCCA cells, identifying a regulatory mechanism of CXCL12 secretion. In addition, immunohistochemistry analysis showed that the highest expression of CXCL12 in stromal fibroblasts and the least levels of TGF-β in iCCA cells are found at the invasive front of the tumor, probably favoring CCA invasion [82]. On the other hand, CXCL12 is positively modulated by angiotensin II in HSCs, and in turn CXCL12 increases activation and proliferation of HSCs, acting in autocrine fashion. In cancer cells, this chemokine induces EMT transition, invasion and migration. Notably, in human iCCA, CXCR4 is also expressed in fibroblast-like stromal cells [83]. The effect of the autocrine action of CXCL12 in CCA was recently highlighted in a study where high CXCL12 expression was associated with metastasis and poor prognosis, and CXCL12 knockdown in CCA cells reduced their migration and invasion, but not proliferation [84].

CXCL12/CXCR4 signaling in CCA cells has also been explored. ERK1/2 and PI3K are involved in CXCL12-induced invasion in pCCA cell lines [120]. More recently, CXCR4 was found upregulated in cancer cells expressing CD24, a cell surface protein associated with adverse prognosis of CCA, together with p-ERK1/2 [120]. Our research group reported that, upon CXCR4 activation, Akt and ERK show an oscillatory pattern of phosphorylation, similarly to that observed in other cancer cells. Moreover, we showed that CXCR4 expression was remarkably higher in iCCA human tissues, compared to non-neoplastic tissues and detected CXCL12 in HSCs conditioned medium capable to promote migration of iCCA cells [71].

CXCL12/CXCR4 interaction can also induce activation of the canonical Wnt pathway that plays a key role in iCCA growth, metastasis and cancer susceptibility. Moreover, CXCR4 levels closely correlate with tumor progression, metastasis and lower overall survival. CXCR4-depleted cells showed reduced growth and reduced tumorigenesis in mice xenografts [121].

CXCR4 expression is also upregulated in eCCA human tissues, with positive correlation with lymph node metastasis and neural invasion [122]. The same group later reported an interaction between metastasis-associated lung adenocarcinoma transcript 1 (MALAT1) and miR-204, which affects human eCCA growth and invasion directly targeting CXCR4. MALAT1 levels were higher in eCCA tissues than in surrounding non-tumor areas and negatively correlated with survival of eCCA patients [123].

A recent study reported an inverse correlation between the levels of osteopontin (OPN) and CXCR4. Low levels of circulating OPN were associated with aggressive characteristics, poor prognosis and low efficacy of chemotherapy in iCCA patients. OPN could inhibit the aggressiveness of cancer cells by negatively regulating metalloproteinase (MMP)1, MMP10, and CXCR4 [124].

A recent study, conducted by our group, has focused on the role of CXCL12/CXCR7 interaction in CCA. We found that CXCR7 was overexpressed in iCCA cells and analyzed its role using two CXCR7 antagonists and genetic interference. The CXCL12/CXCR7 axis was found to regulate adhesion, migration, invasion, survival and growth of iCCA cells. Noteworthy, the expression of CXCR7 was higher in CCA stem-like cells, and CXCR7 down-regulation reduced the ability to form stem-like cell enriched spheres, supporting the relevance of CXCR7 in tumor aggressiveness [85].

The above-illustrated data suggest the possible role of the CXCL12/CXCR4/CXCR7 axis as therapeutic target. Some inhibitors of CXCR4, like AMD3100, have been widely used in CCA studies [71,119,120]. They have been approved by the FDA and are clinically feasible [125]. Recently pharmacological inhibition of CXCL12/CXCR4 axis has been combined to anti-PD-L1 immunotherapy [126,127]. Along these lines, Xie et al. [128] developed PCX polymers from either AMD3100 or novel CXCR4-inhibiting monocyclam inhibitors that, besides affecting CXCR4/CXCL12 axis, introduce nucleic acids into the cancer cells to enhance the anticancer efficacy. The combined treatment, consisting of PCX and miR-200c, synergistically inhibited CCA cell migration, due to CXCR4 blockade and EMT prevention.

## 8. CCL2/CCR2

CCL2 exerts a key role in inflammatory reactions, promoting extravasation of a high variety of immune cells [129,130,131]. Several cells secrete CCL2, either constitutively or upon stimulation, including monocytes, smooth muscle cells, fibroblasts, epithelial and endothelial cells [131,132]. CCL2 acts through the CCR2 receptor [133], which exists in two alternative spliced forms, CCR2A and CCR2B, differing in the C-terminal tails [134]. CCR2A is expressed by mononuclear cells and vascular smooth muscle cells [135], whereas CCR2B is predominantly expressed in monocytes and activated NK cells [135].

CCL2 can also interact with the atypical chemokine receptors ACKR1 and ACKR2 [136], that are mainly expressed by non-leukocyte cell types [136,137] and regulate chemokine gradients [136,137].

As a mediator of inflammation, CCL2 recruits inflammatory cells, modifying the environment in which cancer eventually develops.

CCL2 plays a crucial role in the pathogenesis of liver disease, not only mediating inflammation but also favoring the activation of pro-fibrogenic cells [138]. In the chronically inflamed liver, the accumulation of monocyte-derived macrophages, recruited by CCL2, represents a critical event in the angiogenic and fibrogenic process that may lead to tumor development [139]. Current evidence shows that CCL2 is involved in tumor initiation and progression, by mediating monocyte recruitment/maturation into TAMs, as well as acting on stromal and tumor cells to modulate angiogenesis, metastasis and cancer cell proliferation [92]. Andersen et al. [69] found altered CCL2 expression in the microenvironment of human iCCA specimens, where a stromal signature characterized by upregulation of IL-6 and TGF-β3, in association with poor prognosis, was identified. Lin et al. [140] have shown that CAFs, isolated from samples of iCCA patients, are the major source for CCL2. Moreover, fibroblast activation protein (FAP), a serine protease selectively expressed by CAFs in solid tumors [141], was also expressed in CAFs from iCCA patients, where induced STAT3 activation and CCL2 secretion, greatly reduced by FAP knockdown [140]. In vitro studies revealed that FAP has a critical role in CAFs isolated from iCCA to mediate migration of MDSCs via CCL2 [140]. Finally, in iCCA xenograft model, CCL2 stimulated cell proliferation, induced angiogenic factors (VEGFA, MMP2, MMP9, MMP12, Angiopoietin II) and mediated migration of MDSCs, macrophages [140] and other immune cells, promoting cancer growth [142].

## 9. CCL5/CCR5

CCL5 is widely established as an inflammatory chemokine. CCL5 represents a target gene of NF-κB and is expressed by many different cells, including certain types of tumor cells [143]. CCL5 activity is mediated by CCR1, CCR3 and mainly CCR5 [144]. Upon ligand binding, CCR5 stimulates cell proliferation and survival, glycolysis, immune cell differentiation and growth of progenitor and stem cells [145].

CCL5 recruits several leukocyte subsets towards the injured site, including T cells, macrophages, eosinophils and basophils. In association with cytokines released by T cells, such as IL-2 and IFN-γ, CCL5 induces activation and proliferation of specific NK cells, known as CC chemokine-activated killer cells [143]. Nonetheless, its precise role in tumor development is still unclear. It has been reported that the CCL5/CCR5 axis influences cancer cell proliferation, invasion and the establishment of an immunosuppressive microenvironment [146]. In certain cancer cells CCL5 has been found to promote angiogenesis, through down-regulation of miR-200b via the PI3K/Akt pathway [93]. In bone-marrow-derived human mesenchymal stem cells under inflammatory state (MSC-TI) CCL5 expression was upregulated and increased expression of CCR1, CCR3 and CCR5 was found in CCA cells treated with MSC-TI conditioned medium [147]. Moreover, when either Maraviroc, a CCR5 antagonist [148], or anti-CCL5 antibodies were added to conditioned medium, CCA cell motility was inhibited, suggesting that CCL5 may represent a key factor in influencing CCA biology. Accordingly, when CCA cells were stimulated with CCL5, the chemokine promoted cell migration and invasion, by inducing phosphorylation of Akt together with an increase in MMP2 and MMP9 expression.

## 10. CXCL7/CXCR2

CXCL7 (also known as NAP-2) is a platelet-derived chemokine. It is mainly expressed in platelets as an inactive precursor and its active form is generated at the injured site. CXCL7 belongs to a subset of seven chemokines characterized by a N-terminal ELR motif, which are agonists for CXCR2 [149]. Interacting with CXCR2, CXCL7 functions as a potent chemoattractant and activator of neutrophils [150].

This chemokine participates in diverse cellular processes, such as DNA synthesis, mitosis and glycolysis, and has been implicated in tumor growth [151,152]. It is also implied in different aspects of fibroblast metabolism, such as synthesis of matrix components (hyaluronic acid and glycosaminoglycans) and increase of glucose transporter GLUT1 expression, with consequent glucose uptake [153].

In CCA, CXCL7 has been found to be mostly expressed in tumor tissues in respect to adjacent non-tumor areas, and its overexpression has been associated with a poor prognosis [86]. Both CXCL7 and its receptor are upregulated in intra- and extrahepatic CCA cell lines and sustain cell proliferation and invasion, via Akt. In addition, exposure of CCA cells to CXCL7 enhanced their malignant phenotype. Similar effects were obtained by treating CCA cells with conditioned medium of CXCL7-overexpressing cells, suggesting that this chemokine could modulate CCA features in both autocrine and paracrine manner [86].

## 11. CXCL9/CXCR3

CXCL9 is an ELR-motif negative-CXC chemokine, induced by IFN-γ. It is secreted by different cells, such as macrophages, endothelial cells, hepatocytes and cancer cells [154]. The transcriptional regulation of CXCL9 is a multistep process involving several transcription factors, including STAT1 and NF-κB [155]. CXCL9 is the ligand for CXCR3, through which it mainly acts as chemoattractant for activated immune cells, as T lymphocytes and NK cells. CXCR3 is highly expressed on several immune cells as well as endothelial, epithelial cells and fibroblasts [156,157]. Several studies have demonstrated that abnormal CXCR3 expression is involved in inflammation, angiogenesis, tumor appearance and immune response [158,159,160,161]. In humans three differential spliced forms of CXCR3 have been reported: CXCR3-A, CXCR3-B and CXCR3-alt. CXCR3-A stimulates chemotaxis, proliferation and metastasis, whereas CXCR3-B blocks angiogenesis, migration, cell growth and increases apoptosis, and CXCR3-alt mainly acts as a decoy receptor [162]. Generally, CXCR3 controls many signaling pathways, including MAPK, PLC and PI3K [163,164,165].

Several studies have reported a role for CXCL9 in regulating tumor biology, with controversial results. In a study by Gorbachev et al. [166], CXCL9-deficient fibrosarcoma cells showed higher malignant phenotype than CXCL9-sufficient counterparts. In other reports, CXCL9 promotes tumor growth [167]. The two splicing variants, CXCR3A and CXCR3B, appear to display opposite functions, with pro- and anti-tumor activity, respectively [167,168,169,170]. CXCL9 has been identified as a tumor suppressor, because in 70 iCCA resection specimens high levels of CXCL9 were associated to a favorable postoperative survival. High CXCL9 also correlated with remarkable abundance of tumor-infiltrating NK cells [87]. The protective role of CXCL9 was confirmed in a mouse model of iCCA, in which CXCL9 knockdown led to greater tumor masses, affecting NK cell recruitment into tumor areas. Fukuda et al. also investigated the impact of CXCL9 on tumor cell biological properties in vitro, demonstrating that CXCL9 was released in response to inflammatory stimuli by CCA cell lines. Moreover, exposure of CCA cells to CXCL9 did not modify either invasion or proliferation rate. These data suggest that boosting the CXCL9 system could represent a promising therapeutic approach, being involved in immunopotentiation [87].

## 12. CCL20/CCR6

CCL20, also known as liver activation regulated chemokine (LARC) or macrophage inflammatory protein-3 (MIP3A) is a small chemokine expressed in several immune cells, including NK [171], neutrophils [172], T helper (Th) 17 cells [173], B-cells [174] as well as in various organs and tissues [175]. As a rare feature among the CC chemokines, CCL20 has an exclusive known receptor, CCR6 [176,177]. CCR6 is expressed in various leukocyte subsets, including CD34+ hematopoietic precursor-derived DCs [178], and memory T [179] and B cells [180]. It can function as an immune mediator, linking immature DCs (iDCs) to adaptive immune response. Once iDCs take up antigen, mature and activate, CCR6 expression is down-regulated and CCR7 up-regulated [181,182,183]. The presence of CCL20 in pro-inflammatory Th17 and regulatory Treg cells suggests that the CCL20/CCR6 axis may regulate both immune activation and suppression [94]. CCL20 exerts a strong chemotactic effect on lymphocytes and a weaker action on neutrophils.

CCL20 has been recently related to CCA. In order to identify shared transcriptional networks in CCA and potential therapeutic targets, Maung et al. [95] analyzed multiple microarray datasets, selecting from Gene Expression Omnibus (GEO) repository [184,185] a number of over-expressed genes, including CCL20, associated with cell cycle, motility and cytokine responsiveness [95]. Exposure of iCCA cells to CCL20 resulted in increased migration and enhanced expression of EMT markers, such as N-cadherin, whereas knockdown of CCR6 reduced CCA cell motility.

These data suggest that targeting CCL20/CCR6 axis could be a new approach for cancer treatment and GSK3050002, a CCL20 neutralizing has been used in a clinical trial to test its safety in human subjects [186].

## 13. CXCL5-CXCR2

CXCL5, also known as epithelial-derived neutrophil-activating peptide-78 (ENA-78) is secreted by inflammatory and endothelial cells of various organs in response to insults [2]. Binding to its receptors, such as CXCR2, which is expressed in neutrophils, monocytes, eosinophils, endothelial cells and others [88], CXCL5 participates in immune cell recruitment, promotes angiogenesis and is involved in tumor progression [187]. In tumors, the CXCL5/CXCR2 axis has been implicated in multiple processes, as angiogenesis, growth, metastasis and chemoresistance, acting on TME, cancer stem cells and immune checkpoints [188].

The biological functions and clinical relevance of CXCL5 in CCA were recently investigated [2], highlighting a role for this chemokine both in CCA cells and TRS. In a study aimed at identifying key proteins involved in the crosstalk between CCA and TME, Okabe et al. [189] employed HSC/iCCA cell co-culture, detecting higher levels of CXCL5 in co-culture medium than in monoculture. CXCL5 was mainly secreted by iCCA cells, on which it exerted a migratory and pro-invasive action, in autocrine manner. Noteworthy, the autocrine loop was predominantly induced by IL-1β-released by HSCs [189].

Immunohistochemistry on human iCCA tissues revealed up-regulated expression of CXCL5 in neoplastic cells, associated with increased expression of α-SMA, a reliable marker of HSCs, and high number of CD66b-expressing neutrophils. Importantly, high levels of CXCL5 correlated with poor overall survival [89]. On the other hand, in xenograft models, injection of CXCL5-depleted CCA cells induced smaller tumors, lesser neutrophil infiltration and lower pulmonary metastasis, than control cells. Indeed, CXCL5 acts as potent chemotactic stimulus for neutrophils in vitro and induces tumoral neutrophil infiltration in vivo through PI3K/Akt and ERK1/2 activation [89].

A recent meta-analysis aimed to evaluate the prognostic significance of CXCL5 in CCA patients, revealed that CXCL5 overexpression was inversely correlated with overall survival [90]. Another recent study, analyzing serum chemokine profiles in CCA, reported that CXCL5 was higher in CCA patients, compared to healthy subjects, and CXCL5 levels correlated with poor prognosis [91]. These reports clearly point at this chemokine as an important player in tumor progression and a possible novel prognostic marker for CCA.

## 14. CX3CL1/CX3CR1

CX3CL1 (also known as fractalkine) [190] is a transmembrane chemokine that mediates leukocyte activation by presenting its chemokine domain (CD) to the cognate receptor, CX3CR1 [191,192]. CX3CL1 can be cleaved by metalloproteinases, such as a disintegrin and metalloproteinase domain-containing protein (ADAM)10 and ADAM17, into a soluble molecule with potent chemoattractant properties [193,194].

Activation of CX3CR1 induces survival pathways in both normal [195] and cancer cells [196]. Moreover, CX3CL1/CX3CR1 signaling triggers a rapid mobilization and accumulation of immune cells to the sites of injury and is involved in several inflammatory diseases [197], such as primary biliary cholangitis (PBC). As a chronic inflammatory milieu represents a common risk factor for the appearance of CCA [198], it is conceivable that this pathway contributes to processes driving bile duct cancer. In a study investigating the role of CX3CL1/CX3CR1 axis in PBC, a condition prompting to CCA [199], CX3CL1 was found over-expressed in injured bile ducts, whereas the number of CX3CR1+ mononuclear cells infiltrated into portal tracts was highly increased [96]. In addition, soluble CX3CL1 was also found to recruit lymphocytes into injured bile ducts in PBC patients [199].

Cellular senescence is known to be involved in the pathophysiology of multiple chronic liver diseases, including hepatocarcinoma (HCC) [97,200]. Senescent cells affect the tissue microenvironment producing senescence-associated secretory phenotypes (SASP), comprising cytokines and chemokines, such as CX3CL1. Senescent BECs of PBC-damaged bile ducts released increased levels of CX3CL1, promoting infiltration of CX3CR1-expressing cells, that further exacerbated bile duct inflammation [201].

Overall, these experimental lines of evidence highlight the possible role of CX3CL1/CX3CR1 axis in the pathogenesis of CCA, through the modulation of inflammatory response. Interestingly, in an in vitro study by Haga et al. [79], CX3CL1 was detected in the secretome of MSCs exposed to CCA cell-derived extravescicles (EVs), although untreated MSCs were unable to release this factor, further sustaining a contribution of this chemokine in CCA.

## 15. Microvescicles

CCA cells also interact with TME through secreted membrane vesicles, small heterogeneous spherical structures containing cytoplasmic components, mRNAs and microRNAs [202], acting as mediators of intercellular communication [203,204]. According to their origin, they are classified in microvesicles and ectosomes, when derive from budding or shedding plasma membranes, and EVs, exosomes and exosome-like vesicles, when originate in intracellular compartments and are secreted by fusion with plasma membrane [205].

These vesicles can interact with local or distant cells by EV-cell membrane contact, fusion or internalization [206], contributing to the development of several diseases, including cancer [207,208]. In tumors, EVs participate in the cross-talk between tumor and stroma, stimulating key processes, as angiogenesis [209], invasion [210], inflammation [79], chemoresistance [211,212] and immune escape [213]. By vesicle release, tumor cells can transfer genetic information, modulating recipient cell behavior [214]. Baj-Krzyworzeka et al. [215] showed that, carrying both proteins and mRNA, tumor cell-derived EVs activate monocytes, increasing the expression of human leukocyte antigen-DR isotype, IL-8, CCL2 and CCL4, production of ROS and secretion of TNF, IL-10 and IL-12p40. In a different study, the same authors reported that, in NOD-SCID mice, Evs, by delivering chemokines, induced angiogenesis and activated monocytes [216].

The involvement of EVs in biliary pathobiology and CCA carcinogenesis was recently described [217]. Haga et al. [79] showed that CCA cell-derived EVs induce the differentiation of bone marrow-derived MSCs into fibroblasts releasing cytokines and chemokines (IL-6, CXCL1 and CCL2), that stimulate cell proliferation via IL-6/STAT3 pathways. Dutta et al. [218] reported that KKU-M213 exosomes promote migration and invasion of H69 cells. Moreover, integrin β4, whose role in CCA is well-established, was recently recognized as an EV component driving future metastatic sites [219].

Along these lines, altered EVs in serum and bile were proposed as diagnostic biomarkers and therapeutic target for CCA [220]. Early diagnosis, non-invasive diagnostic and treatment of CCA is still far from being achievable. The presence of EVs in biological fluids is rendering them an interesting issue in the study of hepatobiliary diseases. Indeed, emerging evidence demonstrated that EVs are key mediators of cancer biology, being also involved in chemoresistance and immune response, suggesting a potential therapeutic application to boost the responsiveness of chemo- and immunotherapy. However, present knowledge of EVs and EV-related chemokines is limited. It is mandatory to in depth study the intrinsic role of EVs in tumor onset, in order to evaluate novel therapeutic strategies for various cancer types, including CCA [221,222].

## 16. Conclusions and Future Perspectives

**Figure 3:**
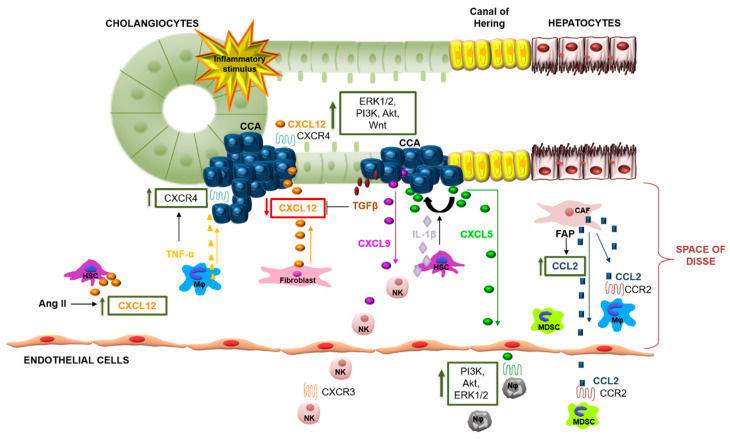
Modulation of CCA development by chemokine networks. Chemokines produced by CCA cells and by intratumor stromal cells, such as CAFs, activate signaling transduction pathways and attract different immune cell types into the CCA TME. CXCL12 released by fibroblast acts on the biological activities of CCA cells. CXCL12 released in CCA microenvironment is negatively modulated by TGF-β and positively by Ang II. TNF-α released by tumor-infiltrating macrophages, promotes the increase of CXCR4 expression in CCA cells. CXCL12/CXCR4 interaction induces activation of PI3K, ERK, Akt and the canonical Wnt signaling in CCA cells. CCL2 secretion, which is induced by FAP in CAFs, mediates migration of MDSCs and macrophages in CCA. CXCL5, secreted by CCA cells and induced by IL-1β released by HSCs, promotes cell migration and invasion through an autocrine loop. In addition, CXCL5 acts as potent chemotactic stimulus for neutrophils inducing neutrophil infiltration through PI3K/Akt and ERK1/2 activation. CXCL9 released by CCA cells correlates with an increase of tumor-infiltrating NK cells. The figure represents a selection among various chemokines involved in CCA progression. Cholangiocarcinoma (CCA), Tumor microenvironment (TME), transforming growth factor-β (TGF-β), angiotensin II (Ang II), tumour necrosis factor-α (TNF-α), phosphoinositide 3-kinase (PI3K), metalloproteinase (MMP), hepatic stellate cell (HSC), cancer-associated fibroblast (CAF), fibroblast activation protein (FAP), myeloid-derived suppressor cell (MDSC), Interleukin-1 β (IL-1β), natural killer cell (NK).

The highly aggressive behavior of CCA, together with its resistance to conventional therapies, mainly accounts for its poor prognosis and the scarce therapeutic options. Chemokines and their receptors are part of a well-orchestrated network, aimed at maintaining host health and to respond to conditions of perturbed environment. Dysregulation of this system results in deleterious responses involved in a variety of diseases, including cancer. Despite extensive knowledge of chemokines in tumor biology (Figure 3), several questions concerning the specific role played by the diverse chemokines in relation of tumor stage during cancer development are still uncertain. A deep understanding of chemokine-induced molecular cascades implicated in CCA biology could be helpful to develop novel approaches to complement surgery and chemotherapy. Moreover, by interfering with pro-inflammatory, -angiogenic and -fibrogenic chemokine pathways, chemokine-based treatments might even contribute to reduce the risk to develop CCA in patients with chronic liver diseases. Understanding the roles of CCA chemokine associated molecular mechanisms could be crucial to identify predictive and prognostic biomarkers.

Recently some chemokines or their receptor antagonists, such as for CXCR4 or CXCR2, have been approved for clinical trials [223,224]; hopefully in the future these studies could be extended also to cholangiocarcinoma.

Indeed, since drugs that selectively induce apoptosis or cytotoxicity in CAFs or TAMs are of great interest, targeting CCA stromal population for therapeutic purposes has been recently proposed [75,225]. Pharmaceutical approaches targeting chemokine pathways might be a strategy to treat CCA in a complementary way with conventional surgery and chemotherapy. Thus, pharmacological agents able to reduce cancer cell aggressiveness could be part of neoadjuvant strategies, to halt tumor dissemination before surgery, or even during palliative treatments to slow the development of the tumor.

However, CCA therapeutic approaches have given to date unsatisfactory results, due to the high tumor heterogeneity that underlies activation of different signaling cascades. Thus, more accurate pharmacological treatments should be selectively designated to improve patient’s outcome.

In addition, CCA preclinical studies performed so far are limited due to poor animal models capable to mirror clinical and genetic features of the human disease. Indeed, besides xenograft model that are not specific for CCA, there are several transgenic mouse models, which can be eligible to study CCA developmental mechanisms. However, these models own technical difficulties and are very expensive. Therefore, innovative and well-defined molecular models of CCA are required for preclinical intervention trials.

## Figures and Tables

**Table 1:** Major chemokines and their receptors involved in CCA biology.

Chemokine Family	Chemokine	Chemokine Receptor	Target Cells	Key Functions	References
CXCL	CXCL12	CXCR4	CAFs CCA cells	CCA cell survival, migration and invasion; EMT transition; metastasis; poor prognosis	[71,82,83,84]
		CXCR7	CCA cells	CCA cell adhesion, migration, invasion, growth and survival	[85]
	CXCL7	CXCR2	CCA cells Fibroblasts Immune cells	CCA cell proliferation and invasion; poor prognosis	[86]
	CXCL9	CXCR3	CCA cells Immune cells Fibroblasts	Inflammation	[87]
	CXCL5	CXCR2	CCA cells Neutrophils	CCA cell migration and invasion; poor prognosis; neutrophil infiltration	[88,89,90,91]
CCL	CCL2	CCR2	Monocytes, Macrophages MDCs	Immune cell migration; poor prognosis	[77,92]
	CCL5	CCR5	CCA cells Immune cells Stem cells	CCA cell migration and invasion	[93]
	CCL20	CCR6	CCA cells Immune cells	CCA cell migration; EMT transition	[94,95]
CX3CL	CX3CL1	CX3CR1	Mononuclear cells	Infiltration of immune cells; inflammation	[96,97]

## Abbreviations

Cancer-associated fibroblasts (CAFs); cholangiocarcinoma (CCA); extracellular matrix (ECM); hepatic stellate cells (HSCs); tumor-associated macrophages (TAMs); tumor microenvironment (TME).

## References

[1] Gentilini A., Pastore M., Marra F., Raggi C. (2018). The Role of Stroma in Cholangiocarcinoma: The Intriguing Interplay between Fibroblastic Component, Immune Cell Subsets and Tumor Epithelium. Int. J. Mol. Sci..

[2] Chen Z., Guo P., Xie X., Yu H., Wang Y., Chen G. (2019). The role of tumour microenvironment: A new vision for cholangiocarcinoma. J. Cell Mol. Med..

[3] Pejin B., Jovanović K.K., Mojović M., Savić A.G. (2013). New and highly potent antitumor natural products from marine-derived fungi: Covering the period from 2003 to 2012. Curr. Top. Med. Chem..

[4] Brivio S., Cadamuro M., Strazzabosco M., Fabris L. (2017). Tumor reactive stroma in cholangiocarcinoma: The fuel behind cancer aggressiveness. World J. Hepatol..

[5] Sirica A.E., Gores G.J., Groopman J.D., Selaru F.M., Strazzabosco M., Wei Wang X., Zhu A.X. (2019). Intrahepatic Cholangiocarcinoma: Continuing Challenges and Translational Advances. Hepatology.

[6] Cadamuro M., Brivio S., Spirli C., Joplin R.E., Strazzabosco M., Fabris L. (2017). Autocrine and Paracrine Mechanisms Promoting Chemoresistance in Cholangiocarcinoma. Int. J. Mol. Sci..

[7] Sirica A.E. (2011). The role of cancer-associated myofibroblasts in intrahepatic cholangiocarcinoma. Nat. Rev. Gastroenterol. Hepatol..

[8] Roy S., Glaser S., Chakraborty S. (2019). Inflammation and Progression of Cholangiocarcinoma: Role of Angiogenic and Lymphangiogenic Mechanisms. Front. Med..

[9] Kasper H.U., Drebber U., Stippel D.L., Dienes H.P., Gillessen A. (2009). Liver tumor infiltrating lymphocytes: Comparison of hepatocellular and cholangiolar carcinoma. World J. Gastroenterol..

[10] Goeppert B., Frauenschuh L., Zucknick M., Stenzinger A., Andrulis M., Klauschen F., Joehrens K., Warth A., Renner M., Mehrabi A. (2013). Prognostic impact of tumour-infiltrating immune cells on biliary tract cancer. Br. J. Cancer.

[11] Jung I.H., Kim D.H., Yoo D.K., Baek S.Y., Jeong S.H., Jung D.E., Park S.W., Chung Y.Y. (2018). Study of Natural Killer (NK) Cell Cytotoxicity Against Cholangiocarcinoma in a Nude Mouse Model. In Vivo.

[12] Morisaki T., Umebayashi M., Kiyota A., Koya N., Tanaka H., Onishi H., Katano M. (2012). Combining cetuximab with killer lymphocytes synergistically inhibits human cholangiocarcinoma cells in vitro. Anticancer Res..

[13] Jonuleit H., Schmitt E., Schuler G., Knop J., Enk A.H. (2000). Induction of interleukin 10-producing, nonproliferating CD4(+) T cells with regulatory properties by repetitive stimulation with allogeneic immature human dendritic cells. J. Exp. Med..

[14] Tan D.W., Fu Y., Su Q., Guan M.J., Kong P., Wang S.Q., Wang H.L. (2016). Prognostic Significance of Neutrophil to Lymphocyte Ratio in Oncologic Outcomes of Cholangiocarcinoma: A Meta-analysis. Sci. Rep..

[15] Fabris L., Alvaro D. (2012). The prognosis of perihilar cholangiocarcinoma after radical treatments. Hepatology.

[16] Sha M., Jeong S., Wang X., Tong Y., Cao J., Sun H.Y., Xia L., Xu N., Xi Z.F., Zhang J.J. (2019). Tumor-associated lymphangiogenesis predicts unfavorable prognosis of intrahepatic cholangiocarcinoma. BMC Cancer.

[17] Xiao K., Ouyang Z., Tang H.H. (2018). Inhibiting the proliferation and metastasis of hilar cholangiocarcinoma cells by blocking the expression of vascular endothelial growth factor with small interfering RNA. Oncol. Lett..

[18] Zlotnik A., Yoshie O. (2000). Chemokines: A new classification system and their role in immunity. Immunity.

[19] Raggi C., Correnti M., Sica A., Andersen J.B., Cardinale V., Alvaro D., Chiorino G., Forti E., Glaser S., Alpini G. (2017). Cholangiocarcinoma stem-like subset shapes tumor-initiating niche by educating associated macrophages. J. Hepatol..

[20] Wescott M.P., Kufareva I., Paes C., Goodman J.R., Thaker Y., Puffer B.A., Berdougo E., Rucker J.B., Handel T.M., Doranz B.J. (2016). Signal transmission through the CXC chemokine receptor 4 (CXCR4) transmembrane helices. Proc. Natl. Acad. Sci. USA.

[21] Bachelerie F., Graham G.J., Locati M., Mantovani A., Murphy P.M., Nibbs R., Rot A., Sozzani S., Thelen M. (2014). New nomenclature for atypical chemokine receptors. Nat. Immunol..

[22] Stone M.J., Hayward J.A., Huang C., Huma Z.E., Sanchez J. (2017). Mechanisms of Regulation of the Chemokine-Receptor Network. Int. J. Mol. Sci..

[23] Vacchini A., Busnelli M., Chini B., Locati M., Borroni E.M. (2016). Analysis of G Protein and β-Arrestin Activation in Chemokine Receptors Signaling. Methods Enzymol..

[24] Bonecchi R., Savino B., Borroni E.M., Mantovani A., Locati M. (2010). Chemokine decoy receptors: Structure-function and biological properties. Curr. Top. Microbiol. Immunol..

[25] Borroni E.M., Savino B., Bonecchi R., Locati M. (2018). Chemokines sound the alarmin: The role of atypical chemokine in inflammation and cancer. Semin. Immunol..

[26] Le Y., Zhou Y., Iribarren P., Wang J. (2004). Chemokines and chemokine receptors: Their manifold roles in homeostasis and disease. Cell Mol. Immunol..

[27] Chen K., Liu M., Liu Y., Wang C., Yoshimura T., Gong W., Le Y., Tessarollo L., Wang J.M. (2013). Signal relay by CC chemokine receptor 2 (CCR2) and formylpeptide receptor 2 (Fpr2) in the recruitment of monocyte-derived dendritic cells in allergic airway inflammation. J. Biol. Chem..

[28] Graham G.J. (2009). D6 and the atypical chemokine receptor family: Novel regulators of immune and inflammatory processes. Eur. J. Immunol..

[29] Zlotnik A., Burkhardt A.M., Homey B. (2011). Homeostatic chemokine receptors and organ-specific metastasis. Nat. Rev. Immunol..

[30] Proudfoot A.E.I., Johnson Z., Bonvin P., Handel T.M. (2017). Glycosaminoglycan Interactions with Chemokines Add Complexity to a Complex System. Pharmaceuticals.

[31] Wang X., Sharp J.S., Handel T.M., Prestegard J.H. (2013). Chemokine oligomerization in cell signaling and migration. Prog. Mol. Biol. Transl. Sci..

[32] Mortier A., Van Damme J., Proost P. (2008). Regulation of chemokine activity by posttranslational modification. Pharmacol. Ther..

[33] Starr A.E., Dufour A., Maier J., Overall C.M. (2012). Biochemical analysis of matrix metalloproteinase activation of chemokines CCL15 and CCL23 and increased glycosaminoglycan binding of CCL16. J. Biol. Chem..

[34] Mortier A., Van Damme J., Proost P. (2012). Overview of the mechanisms regulating chemokine activity and availability. Immunol. Lett..

[35] Bronger H., Karge A., Dreyer T., Zech D., Kraeft S., Avril S., Kiechle M., Schmitt M. (2017). Induction of cathepsin B by the CXCR3 chemokines CXCL9 and CXCL10 in human breast cancer cells. Oncol. Lett..

[36] Barreira da Silva R., Laird M.E., Yatim N., Fiette L., Ingersoll M.A., Albert M.L. (2015). Dipeptidylpeptidase 4 inhibition enhances lymphocyte trafficking, improving both naturally occurring tumor immunity and immunotherapy. Nat. Immunol..

[37] Bronger H., Magdolen V., Goettig P., Dreyer T. (2019). Proteolytic chemokine cleavage as a regulator of lymphocytic infiltration in solid tumors. Cancer Metastasis Rev..

[38] Sarvaiya P.J., Guo D., Ulasov I., Gabikian P., Lesniak M.S. (2013). Chemokines in tumor progression and metastasis. Oncotarget.

[39] Richmond A. (2002). Nf-kappa B, chemokine gene transcription and tumour growth. Nat. Rev. Immunol..

[40] Schioppa T., Uranchimeg B., Saccani A., Biswas S.K., Doni A., Rapisarda A., Bernasconi S., Saccani S., Nebuloni M., Vago L. (2003). Regulation of the chemokine receptor CXCR4 by hypoxia. J. Exp. Med..

[41] Staller P., Sulitkova J., Lisztwan J., Moch H., Oakeley E.J., Krek W. (2003). Chemokine receptor CXCR4 downregulated by von Hippel-Lindau tumour suppressor pVHL. Nature.

[42] Végran F., Boidot R., Michiels C., Sonveaux P., Feron O. (2011). Lactate influx through the endothelial cell monocarboxylate transporter MCT1 supports an NF-κB/IL-8 pathway that drives tumor angiogenesis. Cancer Res..

[43] Pelicano H., Lu W., Zhou Y., Zhang W., Chen Z., Hu Y., Huang P. (2009). Mitochondrial dysfunction and reactive oxygen species imbalance promote breast cancer cell motility through a CXCL14-mediated mechanism. Cancer Res..

[44] Zhou Y., Larsen P.H., Hao C., Yong V.W. (2002). CXCR4 is a major chemokine receptor on glioma cells and mediates their survival. J. Biol. Chem..

[45] Barbero S., Bonavia R., Bajetto A., Porcile C., Pirani P., Ravetti J.L., Zona G.L., Spaziante R., Florio T., Schettini G. (2003). Stromal cell-derived factor 1alpha stimulates human glioblastoma cell growth through the activation of both extracellular signal-regulated kinases 1/2 and Akt. Cancer Res..

[46] Murakami T., Cardones A.R., Finkelstein S.E., Restifo N.P., Klaunberg B.A., Nestle F.O., Castillo S.S., Dennis P.A., Hwang S.T. (2003). Immune evasion by murine melanoma mediated through CC chemokine receptor-10. J. Exp. Med..

[47] Lu P., Nakamoto Y., Nemoto-Sasaki Y., Fujii C., Wang H., Hashii M., Ohmoto Y., Kaneko S., Kobayashi K., Mukaida N. (2003). Potential interaction between CCR1 and its ligand, CCL3, induced by endogenously produced interleukin-1 in human hepatomas. Am. J. Pathol..

[48] Mañes S., Mira E., Colomer R., Montero S., Real L.M., Gómez-Moutón C., Jiménez-Baranda S., Garzón A., Lacalle R.A., Harshman K. (2003). CCR5 expression influences the progression of human breast cancer in a p53-dependent manner. J. Exp. Med..

[49] Bates R.C., DeLeo M.J., Mercurio A.M. (2004). The epithelial-mesenchymal transition of colon carcinoma involves expression of IL-8 and CXCR-1-mediated chemotaxis. Exp. Cell Res..

[50] Bertran E., Caja L., Navarro E., Sancho P., Mainez J., Murillo M.M., Vinyals A., Fabra A., Fabregat I. (2009). Role of CXCR4/SDF-1 alpha in the migratory phenotype of hepatoma cells that have undergone epithelial-mesenchymal transition in response to the transforming growth factor-beta. Cell Signal..

[51] Hwang W.L., Yang M.H., Tsai M.L., Lan H.Y., Su S.H., Chang S.C., Teng H.W., Yang S.H., Lan Y.T., Chiou S.H. (2011). SNAIL regulates interleukin-8 expression, stem cell-like activity, and tumorigenicity of human colorectal carcinoma cells. Gastroenterology.

[52] Kiefer F., Siekmann A.F. (2011). The role of chemokines and their receptors in angiogenesis. Cell Mol. Life Sci..

[53] Mehrad B., Keane M.P., Strieter R.M. (2007). Chemokines as mediators of angiogenesis. Thromb. Haemost..

[54] Burger J.A., Tsukada N., Burger M., Zvaifler N.J., Dell’Aquila M., Kipps T.J. (2000). Blood-derived nurse-like cells protect chronic lymphocytic leukemia B cells from spontaneous apoptosis through stromal cell-derived factor-1. Blood.

[55] Lazennec G., Richmond A. (2010). Chemokines and chemokine receptors: New insights into cancer-related inflammation. Trends Mol. Med..

[56] Singh S., Wu S., Varney M., Singh A.P., Singh R.K. (2011). CXCR1 and CXCR2 silencing modulates CXCL8-dependent endothelial cell proliferation, migration and capillary-like structure formation. Microvasc. Res..

[57] Lewis C.E., Pollard J.W. (2006). Distinct role of macrophages in different tumor microenvironments. Cancer Res..

[58] Mantovani A., Schioppa T., Porta C., Allavena P., Sica A. (2006). Role of tumor-associated macrophages in tumor progression and invasion. Cancer Metastasis Rev..

[59] Sozzani S., Rusnati M., Riboldi E., Mitola S., Presta M. (2007). Dendritic cell-endothelial cell cross-talk in angiogenesis. Trends Immunol..

[60] Rehman J., Landman J., Sundaram C., Clayman R.V. (2003). Tissue chemoablation. J. Endourol..

[61] Wolf M.J., Hoos A., Bauer J., Boettcher S., Knust M., Weber A., Simonavicius N., Schneider C., Lang M., Stürzl M. (2012). Endothelial CCR2 signaling induced by colon carcinoma cells enables extravasation via the JAK2-Stat5 and p38MAPK pathway. Cancer Cell.

[62] Halvorsen E.C., Hamilton M.J., Young A., Wadsworth B.J., LePard N.E., Lee H.N., Firmino N., Collier J.L., Bennewith K.L. (2016). Maraviroc decreases CCL8-mediated migration of CCR5(+) regulatory T cells and reduces metastatic tumor growth in the lungs. Oncoimmunology.

[63] Kitamura T., Pollard J.W. (2015). Therapeutic potential of chemokine signal inhibition for metastatic breast cancer. Pharmacol. Res..

[64] Verbeke H., Struyf S., Laureys G., Van Damme J. (2011). The expression and role of CXC chemokines in colorectal cancer. Cytokine Growth Factor Rev..

[65] Haider C., Hnat J., Wagner R., Huber H., Timelthaler G., Grubinger M., Coulouarn C., Schreiner W., Schlangen K., Sieghart W. (2019). Transforming Growth Factor-β and Axl Induce CXCL5 and Neutrophil Recruitment in Hepatocellular Carcinoma. Hepatology.

[66] Najjar Y.G., Rayman P., Jia X., Pavicic P.G., Rini B.I., Tannenbaum C., Ko J., Haywood S., Cohen P., Hamilton T. (2017). Myeloid-Derived Suppressor Cell Subset Accumulation in Renal Cell Carcinoma Parenchyma Is Associated with Intratumoral Expression of IL1β, IL8, CXCL5, and Mip-1α. Clin. Cancer Res..

[67] Lecot P., Sarabi M., Pereira Abrantes M., Mussard J., Koenderman L., Caux C., Bendriss-Vermare N., Michallet M.C. (2019). Neutrophil Heterogeneity in Cancer: From Biology to Therapies. Front. Immunol..

[68] Granot Z. (2019). Neutrophils as a Therapeutic Target in Cancer. Front. Immunol..

[69] Andersen J.B., Spee B., Blechacz B.R., Avital I., Komuta M., Barbour A., Conner E.A., Gillen M.C., Roskams T., Roberts L.R. (2012). Genomic and genetic characterization of cholangiocarcinoma identifies therapeutic targets for tyrosine kinase inhibitors. Gastroenterology.

[70] Heits N., Heinze T., Bernsmeier A., Kerber J., Hauser C., Becker T., Kalthoff H., Egberts J.H., Braun F. (2016). Influence of mTOR-inhibitors and mycophenolic acid on human cholangiocellular carcinoma and cancer associated fibroblasts. BMC Cancer.

[71] Gentilini A., Rombouts K., Galastri S., Caligiuri A., Mingarelli E., Mello T., Marra F., Mantero S., Roncalli M., Invernizzi P. (2012). Role of the stromal-derived factor-1 (SDF-1)-CXCR4 axis in the interaction between hepatic stellate cells and cholangiocarcinoma. J. Hepatol..

[72] Okabe H., Beppu T., Hayashi H., Horino K., Masuda T., Komori H., Ishikawa S., Watanabe M., Takamori H., Iyama K. (2009). Hepatic stellate cells may relate to progression of intrahepatic cholangiocarcinoma. Ann. Surg. Oncol..

[73] Claperon A., Mergey M., Aoudjehane L., Ho-Bouldoires T.H., Wendum D., Prignon A., Merabtene F., Firrincieli D., Desbois-Mouthon C., Scatton O. (2013). Hepatic myofibroblasts promote the progression of human cholangiocarcinoma through activation of epidermal growth factor receptor. Hepatology.

[74] Okabe H., Beppu T., Hayashi H., Ishiko T., Masuda T., Otao R., Horlad H., Jono H., Ueda M., Shinriki S. (2011). Hepatic stellate cells accelerate the malignant behavior of cholangiocarcinoma cells. Ann. Surg. Oncol..

[75] Mertens J.C., Fingas C.D., Christensen J.D., Smoot R.L., Bronk S.F., Werneburg N.W., Gustafson M.P., Dietz A.B., Roberts L.R., Sirica A.E. (2013). Therapeutic effects of deleting cancer-associated fibroblasts in cholangiocarcinoma. Cancer Res..

[76] Cadamuro M., Morton S.D., Strazzabosco M., Fabris L. (2013). Unveiling the role of tumor reactive stroma in cholangiocarcinoma: An opportunity for new therapeutic strategies. Transl. Gastrointest. Cancer.

[77] Yang X., Lin Y., Shi Y., Li B., Liu W., Yin W., Dang Y., Chu Y., Fan J., He R. (2016). FAP Promotes Immunosuppression by Cancer-Associated Fibroblasts in the Tumor Microenvironment via STAT3-CCL2 Signaling. Cancer Res..

[78] Sica A., Invernizzi P., Mantovani A. (2014). Macrophage plasticity and polarization in liver homeostasis and pathology. Hepatology.

[79] Haga H., Yan I.K., Takahashi K., Wood J., Zubair A., Patel T. (2015). Tumour cell-derived extracellular vesicles interact with mesenchymal stem cells to modulate the microenvironment and enhance cholangiocarcinoma growth. J. Extracell. Vesicles.

[80] Wang W., Zhong W., Yuan J., Yan C., Hu S., Tong Y., Mao Y., Hu T., Zhang B., Song G. (2015). Involvement of Wnt/β-catenin signaling in the mesenchymal stem cells promote metastatic growth and chemoresistance of cholangiocarcinoma. Oncotarget.

[81] Chen F., Zhuang X., Lin L., Yu P., Wang Y., Shi Y., Hu G., Sun Y. (2015). New horizons in tumor microenvironment biology: Challenges and opportunities. BMC Med..

[82] Ohira S., Itatsu K., Sasaki M., Harada K., Sato Y., Zen Y., Ishikawa A., Oda K., Nagasaka T., Nimura Y. (2006). Local balance of transforming growth factor-beta1 secreted from cholangiocarcinoma cells and stromal-derived factor-1 secreted from stromal fibroblasts is a factor involved in invasion of cholangiocarcinoma. Pathol. Int..

[83] Okamoto K., Tajima H., Nakanuma S., Sakai S., Makino I., Kinoshita J., Hayashi H., Nakamura K., Oyama K., Nakagawara H. (2012). Angiotensin II enhances epithelial-to-mesenchymal transition through the interaction between activated hepatic stellate cells and the stromal cell-derived factor-1/CXCR4 axis in intrahepatic cholangiocarcinoma. Int. J. Oncol..

[84] Miyata T., Yamashita Y.I., Yoshizumi T., Shiraishi M., Ohta M., Eguchi S., Aishima S., Fujioka H., Baba H. (2019). CXCL12 expression in intrahepatic cholangiocarcinoma is associated with metastasis and poor prognosis. Cancer Sci..

[85] Gentilini A., Caligiuri A., Raggi C., Rombouts K., Pinzani M., Lori G., Correnti M., Invernizzi P., Rovida E., Navari N. (2019). CXCR7 contributes to the aggressive phenotype of cholangiocarcinoma cells. Biochim. Biophys. Acta Mol. Basis Dis..

[86] Guo Q., Jian Z., Jia B., Chang L. (2017). CXCL7 promotes proliferation and invasion of cholangiocarcinoma cells. Oncol. Rep..

[87] Fukuda Y., Asaoka T., Eguchi H., Yokota Y., Kubo M., Kinoshita M., Urakawa S., Iwagami Y., Tomimaru Y., Akita H. (2020). Endogenous CXCL9 affects prognosis by regulating tumor-infiltrating natural killer cells in intrahepatic cholangiocarcinoma. Cancer Sci..

[88] Veenstra M., Ransohoff R.M. (2012). Chemokine receptor CXCR2: Physiology regulator and neuroinflammation controller?. J. NeuroImmunol..

[89] Zhou S.L., Dai Z., Zhou Z.J., Chen Q., Wang Z., Xiao Y.S., Hu Z.Q., Huang X.Y., Yang G.H., Shi Y.H. (2014). CXCL5 contributes to tumor metastasis and recurrence of intrahepatic cholangiocarcinoma by recruiting infiltrative intratumoral neutrophils. Carcinogenesis.

[90] Hu B., Fan H., Lv X., Chen S., Shao Z. (2018). Prognostic significance of CXCL5 expression in cancer patients: A meta-analysis. Cancer Cell Int..

[91] Lee S.J., Kim J.E., Kim S.T., Lee J., Park S.H., Park J.O., Kang W.K., Park Y.S., Lim H.Y. (2018). The Correlation Between Serum Chemokines and Clinical Outcome in Patients with Advanced Biliary Tract Cancer. Transl. Oncol..

[92] Yoshimura T. (2018). The chemokine MCP-1 (CCL2) in the host interaction with cancer: A foe or ally?. Cell Mol. Immunol..

[93] Liu G.T., Chen H.T., Tsou H.K., Tan T.W., Fong Y.C., Chen P.C., Yang W.H., Wang S.W., Chen J.C., Tang C.H. (2014). CCL5 promotes VEGF-dependent angiogenesis by down-regulating miR-200b through PI3K/Akt signaling pathway in human chondrosarcoma cells. Oncotarget.

[94] Comerford I., Bunting M., Fenix K., Haylock-Jacobs S., Litchfield W., Harata-Lee Y., Turvey M., Brazzatti J., Gregor C., Nguyen P. (2010). An immune paradox: How can the same chemokine axis regulate both immune tolerance and activation?: CCR6/CCL20: A chemokine axis balancing immunological tolerance and inflammation in autoimmune disease. Bioessays.

[95] Maung H.M.W., Chan-On W., Kunkeaw N., Khaenam P. (2020). Common transcriptional programs and the role of chemokine (CC motif) ligand 20 (CCL20) in cell migration of cholangiocarcinoma. EXCLI J..

[96] Isse K., Harada K., Zen Y., Kamihira T., Shimoda S., Harada M., Nakanuma Y. (2005). Fractalkine and CX3CR1 are involved in the recruitment of intraepithelial lymphocytes of intrahepatic bile ducts. Hepatology.

[97] Sasaki M., Ikeda H., Sato Y., Nakanuma Y. (2006). Decreased expression of Bmi1 is closely associated with cellular senescence in small bile ducts in primary biliary cirrhosis. Am. J. Pathol..

[98] Zhou Y., Cao H.B., Li W.J., Zhao L. (2018). The CXCL12 (SDF-1)/CXCR4 chemokine axis: Oncogenic properties, molecular targeting, and synthetic and natural product CXCR4 inhibitors for cancer therapy. Chin. J. Nat. Med..

[99] Nagarsheth N., Wicha M.S., Zou W. (2017). Chemokines in the cancer microenvironment and their relevance in cancer immunotherapy. Nat. Rev. Immunol..

[100] Nazari A., Khorramdelazad H., Hassanshahi G. (2017). Biological/pathological functions of the CXCL12/CXCR4/CXCR7 axes in the pathogenesis of bladder cancer. Int. J. Clin. Oncol..

[101] Schulz O., Hammerschmidt S.I., Moschovakis G.L., Förster R. (2016). Chemokines and Chemokine Receptors in Lymphoid Tissue Dynamics. Annu. Rev. Immunol..

[102] Gagliardi F., Narayanan A., Reni M., Franzin A., Mazza E., Boari N., Bailo M., Zordan P., Mortini P. (2014). The role of CXCR4 in highly malignant human gliomas biology: Current knowledge and future directions. Glia.

[103] Burger J.A., Kipps T.J. (2006). CXCR4: A key receptor in the crosstalk between tumor cells and their microenvironment. Blood.

[104] Mortezaee K. (2020). CXCL12/CXCR4 axis in the microenvironment of solid tumors: A critical mediator of metastasis. Life Sci..

[105] Ding Y., Du Y. (2019). Clinicopathological significance and prognostic role of chemokine receptor CXCR4 expression in pancreatic ductal adenocarcinoma, a meta-analysis and literature review. Int. J. Surg..

[106] Yang J., Zhang L., Jiang Z., Ge C., Zhao F., Jiang J., Tian H., Chen T., Xie H., Cui Y. (2019). TCF12 promotes the tumorigenesis and metastasis of hepatocellular carcinoma via upregulation of CXCR4 expression. Theranostics.

[107] Wald O. (2018). CXCR4 Based Therapeutics for Non-Small Cell Lung Cancer (NSCLC). J. Clin. Med..

[108] Zheng N., Liu W., Chen J., Li B., Liu J., Wang J., Gao Y., Shao J., Jia L. (2019). CXCR7 is not obligatory for CXCL12-CXCR4-induced epithelial-mesenchymal transition in human ovarian cancer. Mol. Carcinog..

[109] Coniglio S.J. (2018). Role of Tumor-Derived Chemokines in Osteolytic Bone Metastasis. Front. Endocrinol..

[110] Hu Y., Zang J., Qin X., Yan D., Cao H., Zhou L., Ni J., Yu S., Wu J., Feng J.F. (2017). Epithelial-to-mesenchymal transition correlates with gefitinib resistance in NSCLC cells and the liver X receptor ligand GW3965 reverses gefitinib resistance through inhibition of vimentin. OncoTargets Ther..

[111] De Luca A., D’Alessio A., Gallo M., Maiello M.R., Bode A.M., Normanno N. (2014). Src and CXCR4 are involved in the invasiveness of breast cancer cells with acquired resistance to lapatinib. Cell Cycle.

[112] Floranović M.P., Veličković L.J. (2019). Effect of CXCL12 and Its Receptors on Unpredictable Renal Cell Carcinoma. Clin. Genitourin. Cancer.

[113] Burns J.M., Summers B.C., Wang Y., Melikian A., Berahovich R., Miao Z., Penfold M.E., Sunshine M.J., Littman D.R., Kuo C.J. (2006). A novel chemokine receptor for SDF-1 and I-TAC involved in cell survival, cell adhesion, and tumor development. J. Exp. Med..

[114] Décaillot F.M., Kazmi M.A., Lin Y., Ray-Saha S., Sakmar T.P., Sachdev P. (2011). CXCR7/CXCR4 heterodimer constitutively recruits beta-arrestin to enhance cell migration. J. Biol. Chem..

[115] Levoye A., Balabanian K., Baleux F., Bachelerie F., Lagane B. (2009). CXCR7 heterodimerizes with CXCR4 and regulates CXCL12-mediated G protein signaling. Blood.

[116] Lounsbury N. (2020). Advances in CXCR7 Modulators. Pharmaceuticals.

[117] Hattermann K., Held-Feindt J., Lucius R., Müerköster S.S., Penfold M.E., Schall T.J., Mentlein R. (2010). The chemokine receptor CXCR7 is highly expressed in human glioma cells and mediates antiapoptotic effects. Cancer Res..

[118] Zhao Q., Zhang P., Qin G., Ren F., Zheng Y., Qiao Y., Sun T., Zhang Y. (2018). Role of CXCR7 as a Common Predictor for Prognosis in Solid Tumors: A Meta-Analysis. J. Cancer.

[119] Ohira S., Sasaki M., Harada K., Sato Y., Zen Y., Isse K., Kozaka K., Ishikawa A., Oda K., Nimura Y. (2006). Possible regulation of migration of intrahepatic cholangiocarcinoma cells by interaction of CXCR4 expressed in carcinoma cells with tumor necrosis factor-alpha and stromal-derived factor-1 released in stroma. Am. J. Pathol..

[120] Leelawat K., Leelawat S., Narong S., Hongeng S. (2007). Roles of the MEK1/2 and AKT pathways in CXCL12/CXCR4 induced cholangiocarcinoma cell invasion. World J. Gastroenterol..

[121] Zhao S., Wang J., Qin C. (2014). Blockade of CXCL12/CXCR4 signaling inhibits intrahepatic cholangiocarcinoma progression and metastasis via inactivation of canonical Wnt pathway. J. Exp. Clin. Cancer Res..

[122] Tan X.Y., Chang S., Liu W., Tang H.H. (2014). Silencing of CXCR4 inhibits tumor cell proliferation and neural invasion in human hilar cholangiocarcinoma. Gut Liver.

[123] Tan X., Huang Z., Li X. (2017). Long Non-Coding RNA MALAT1 Interacts With miR-204 to Modulate Human Hilar Cholangiocarcinoma Proliferation, Migration, and Invasion by Targeting CXCR4. J. Cell Biochem..

[124] Zhou K.Q., Liu W.F., Yang L.X., Sun Y.F., Hu J., Chen F.Y., Zhou C., Zhang X.Y., Peng Y.F., Yu L. (2019). Circulating osteopontin per tumor volume as a prognostic biomarker for resectable intrahepatic cholangiocarcinoma. Hepatobiliary Surg. Nutr..

[125] Eckert F., Schilbach K., Klumpp L., Bardoscia L., Sezgin E.C., Schwab M., Zips D., Huber S.M. (2018). Potential Role of CXCR4 Targeting in the Context of Radiotherapy and Immunotherapy of Cancer. Front. Immunol..

[126] Chen X., Song E. (2019). Turning foes to friends: Targeting cancer-associated fibroblasts. Nat. Rev. Drug Discov..

[127] Szekely B., Bossuyt V., Li X., Wali V.B., Patwardhan G.A., Frederick C., Silber A., Park T., Harigopal M., Pelekanou V. (2018). Immunological differences between primary and metastatic breast cancer. Ann. Oncol..

[128] Xie Y., Wehrkamp C.J., Li J., Wang Y., Wang Y., Mott J.L., Oupický D. (2016). Delivery of miR-200c Mimic with Poly(amido amine) CXCR4 Antagonists for Combined Inhibition of Cholangiocarcinoma Cell Invasiveness. Mol. Pharm..

[129] Sierra-Filardi E., Nieto C., Domínguez-Soto A., Barroso R., Sánchez-Mateos P., Puig-Kroger A., López-Bravo M., Joven J., Ardavín C., Rodríguez-Fernández J.L. (2014). CCL2 shapes macrophage polarization by GM-CSF and M-CSF: Identification of CCL2/CCR2-dependent gene expression profile. J. Immunol..

[130] Deshmane S.L., Kremlev S., Amini S., Sawaya B.E. (2009). Monocyte chemoattractant protein-1 (MCP-1): An overview. J. Interferon Cytokine Res..

[131] Gschwandtner M., Derler R., Midwood K.S. (2019). More Than Just Attractive: How CCL2 Influences Myeloid Cell Behavior Beyond Chemotaxis. Front. Immunol..

[132] Cushing S.D., Berliner J.A., Valente A.J., Territo M.C., Navab M., Parhami F., Gerrity R., Schwartz C.J., Fogelman A.M. (1990). Minimally modified low density lipoprotein induces monocyte chemotactic protein 1 in human endothelial cells and smooth muscle cells. Proc. Natl. Acad. Sci. USA.

[133] Taylor B.C., Lee C.T., Amaro R.E. (2019). Structural basis for ligand modulation of the CCR2 conformational landscape. Proc. Natl. Acad. Sci. USA.

[134] Charo I.F., Myers S.J., Herman A., Franci C., Connolly A.J., Coughlin S.R. (1994). Molecular cloning and functional expression of two monocyte chemoattractant protein 1 receptors reveals alternative splicing of the carboxyl-terminal tails. Proc. Natl. Acad. Sci. USA.

[135] Bartoli C., Civatte M., Pellissier J.F., Figarella-Branger D. (2001). CCR2A and CCR2B, the two isoforms of the monocyte chemoattractant protein-1 receptor are up-regulated and expressed by different cell subsets in idiopathic inflammatory myopathies. Acta Neuropathol..

[136] Bonecchi R., Graham G.J. (2016). Atypical Chemokine Receptors and Their Roles in the Resolution of the Inflammatory Response. Front. Immunol..

[137] Vacchini A., Locati M., Borroni E.M. (2016). Overview and potential unifying themes of the atypical chemokine receptor family. J. Leukoc. Biol..

[138] Marra F., Tacke F. (2014). Roles for chemokines in liver disease. Gastroenterology.

[139] Tacke F. (2012). Functional role of intrahepatic monocyte subsets for the progression of liver inflammation and liver fibrosis in vivo. Fibrogenesis Tissue Repair.

[140] Lin Y., Li B., Yang X., Cai Q., Liu W., Tian M., Luo H., Yin W., Song Y., Shi Y. (2019). Fibroblastic FAP promotes intrahepatic cholangiocarcinoma growth via MDSCs recruitment. Neoplasia.

[141] Scanlan M.J., Raj B.K., Calvo B., Garin-Chesa P., Sanz-Moncasi M.P., Healey J.H., Old L.J., Rettig W.J. (1994). Molecular cloning of fibroblast activation protein alpha, a member of the serine protease family selectively expressed in stromal fibroblasts of epithelial cancers. Proc. Natl. Acad. Sci. USA.

[142] Ostrand-Rosenberg S., Sinha P. (2009). Myeloid-derived suppressor cells: Linking inflammation and cancer. J. Immunol..

[143] Soria G., Ben-Baruch A. (2008). The inflammatory chemokines CCL2 and CCL5 in breast cancer. Cancer Lett..

[144] Aldinucci D., Colombatti A. (2014). The inflammatory chemokine CCL5 and cancer progression. Mediat. Inflamm..

[145] Jiao X., Nawab O., Patel T., Kossenkov A.V., Halama N., Jaeger D., Pestell R.G. (2019). Recent Advances Targeting CCR5 for Cancer and Its Role in Immuno-Oncology. Cancer Res..

[146] Kershaw M.H., Westwood J.A., Darcy P.K. (2013). Gene-engineered T cells for cancer therapy. Nat. Rev. Cancer.

[147] Zhong W., Tong Y., Li Y., Yuan J., Hu S., Hu T., Song G. (2017). Mesenchymal stem cells in inflammatory microenvironment potently promote metastatic growth of cholangiocarcinoma. Oncotarget.

[148] Ochoa-Callejero L., Pérez-Martínez L., Rubio-Mediavilla S., Oteo J.A., Martínez A., Blanco J.R. (2013). Maraviroc, a CCR5 antagonist, prevents development of hepatocellular carcinoma in a mouse model. PLoS ONE.

[149] Brown A.J., Sepuru K.M., Sawant K.V., Rajarathnam K. (2017). Platelet-Derived Chemokine CXCL7 Dimer Preferentially Exists in the Glycosaminoglycan-Bound Form: Implications for Neutrophil-Platelet Crosstalk. Front. Immunol..

[150] Von Hundelshausen P., Petersen F., Brandt E. (2007). Platelet-derived chemokines in vascular biology. Thromb. Haemost..

[151] Blunk J.A., Sauerstein K., Schmelz M. (2011). Experimental thermal lesions induce beta-thromboglobulin release from activated platelets. Eur. J. Pain.

[152] Grépin R., Guyot M., Giuliano S., Boncompagni M., Ambrosetti D., Chamorey E., Scoazec J.Y., Negrier S., Simonnet H., Pagès G. (2014). The CXCL7/CXCR1/2 axis is a key driver in the growth of clear cell renal cell carcinoma. Cancer Res..

[153] Tai P.K., Liao J.F., Hossler P.A., Castor C.W., Carter-Su C. (1992). Regulation of glucose transporters by connective tissue activating peptide-III isoforms. J. Biol. Chem..

[154] Ding Q., Lu P., Xia Y., Ding S., Fan Y., Li X., Han P., Liu J., Tian D., Liu M. (2016). CXCL9: Evidence and contradictions for its role in tumor progression. Cancer Med..

[155] Hiroi M., Ohmori Y. (2003). The transcriptional coactivator CREB-binding protein cooperates with STAT1 and NF-kappa B for synergistic transcriptional activation of the CXC ligand 9/monokine induced by interferon-gamma gene. J. Biol. Chem..

[156] Loetscher M., Gerber B., Loetscher P., Jones S.A., Piali L., Clark-Lewis I., Baggiolini M., Moser B. (1996). Chemokine receptor specific for IP10 and mig: Structure, function, and expression in activated T-lymphocytes. J. Exp. Med..

[157] Van Raemdonck K., Van den Steen P.E., Liekens S., Van Damme J., Struyf S. (2015). CXCR3 ligands in disease and therapy. Cytokine Growth Factor Rev..

[158] Lu B., Humbles A., Bota D., Gerard C., Moser B., Soler D., Luster A.D., Gerard N.P. (1999). Structure and function of the murine chemokine receptor CXCR3. Eur. J. Immunol..

[159] Zhu G., Yan H.H., Pang Y., Jian J., Achyut B.R., Liang X., Weiss J.M., Wiltrout R.H., Hollander M.C., Yang L. (2015). CXCR3 as a molecular target in breast cancer metastasis: Inhibition of tumor cell migration and promotion of host anti-tumor immunity. Oncotarget.

[160] Groom J.R., Luster A.D. (2011). CXCR3 ligands: Redundant, collaborative and antagonistic functions. Immunol. Cell Biol..

[161] Wadwa M., Klopfleisch R., Adamczyk A., Frede A., Pastille E., Mahnke K., Hansen W., Geffers R., Lang K.S., Buer J. (2016). IL-10 downregulates CXCR3 expression on Th1 cells and interferes with their migration to intestinal inflammatory sites. Mucosal Immunol..

[162] Billottet C., Quemener C., Bikfalvi A. (2013). CXCR3, a double-edged sword in tumor progression and angiogenesis. Biochim. Biophys. Acta.

[163] Bonacchi A., Romagnani P., Romanelli R.G., Efsen E., Annunziato F., Lasagni L., Francalanci M., Serio M., Laffi G., Pinzani M. (2001). Signal transduction by the chemokine receptor CXCR3: Activation of Ras/ERK, Src, and phosphatidylinositol 3-kinase/Akt controls cell migration and proliferation in human vascular pericytes. J. Biol. Chem..

[164] Shahabuddin S., Ji R., Wang P., Brailoiu E., Dun N., Yang Y., Aksoy M.O., Kelsen S.G. (2006). CXCR3 chemokine receptor-induced chemotaxis in human airway epithelial cells: Role of p38 MAPK and PI3K signaling pathways. Am. J. Physiol. Cell Physiol..

[165] Martins V.L., Vyas J.J., Chen M., Purdie K., Mein C.A., South A.P., Storey A., McGrath J.A., O’Toole E.A. (2009). Increased invasive behaviour in cutaneous squamous cell carcinoma with loss of basement-membrane type VII collagen. J. Cell Sci..

[166] Gorbachev A.V., Kobayashi H., Kudo D., Tannenbaum C.S., Finke J.H., Shu S., Farber J.M., Fairchild R.L. (2007). CXC chemokine ligand 9/monokine induced by IFN-gamma production by tumor cells is critical for T cell-mediated suppression of cutaneous tumors. J. Immunol..

[167] Yang C., Zheng W., Du W. (2016). CXCR3A contributes to the invasion and metastasis of gastric cancer cells. Oncol. Rep..

[168] Ding Q., Xia Y., Ding S., Lu P., Sun L., Liu M. (2016). An alternatively spliced variant of CXCR3 mediates the metastasis of CD133+ liver cancer cells induced by CXCL9. Oncotarget.

[169] Wu Q., Dhir R., Wells A. (2012). Altered CXCR3 isoform expression regulates prostate cancer cell migration and invasion. Mol. Cancer.

[170] Datta D., Banerjee P., Gasser M., Waaga-Gasser A.M., Pal S. (2010). CXCR3-B can mediate growth-inhibitory signals in human renal cancer cells by down-regulating the expression of heme oxygenase-1. J. Biol. Chem..

[171] Cella M., Fuchs A., Vermi W., Facchetti F., Otero K., Lennerz J.K., Doherty J.M., Mills J.C., Colonna M. (2009). A human natural killer cell subset provides an innate source of IL-22 for mucosal immunity. Nature.

[172] Scapini P., Laudanna C., Pinardi C., Allavena P., Mantovani A., Sozzani S., Cassatella M.A. (2001). Neutrophils produce biologically active macrophage inflammatory protein-3alpha (MIP-3alpha)/CCL20 and MIP-3beta/CCL19. Eur. J. Immunol..

[173] Yamazaki T., Yang X.O., Chung Y., Fukunaga A., Nurieva R., Pappu B., Martin-Orozco N., Kang H.S., Ma L., Panopoulos A.D. (2008). CCR6 regulates the migration of inflammatory and regulatory T cells. J. Immunol..

[174] Bowman E.P., Campbell J.J., Soler D., Dong Z., Manlongat N., Picarella D., Hardy R.R., Butcher E.C. (2000). Developmental switches in chemokine response profiles during B cell differentiation and maturation. J. Exp. Med..

[175] Schutyser E., Struyf S., Van Damme J. (2003). The CC chemokine CCL20 and its receptor CCR6. Cytokine Growth Factor Rev..

[176] Varona R., Zaballos A., Gutiérrez J., Martín P., Roncal F., Albar J.P., Ardavín C., Márquez G. (1998). Molecular cloning, functional characterization and mRNA expression analysis of the murine chemokine receptor CCR6 and its specific ligand MIP-3alpha. FEBS Lett..

[177] Liao F., Alderson R., Su J., Ullrich S.J., Kreider B.L., Farber J.M. (1997). STRL22 is a receptor for the CC chemokine MIP-3alpha. Biochem. Biophys. Res. Commun..

[178] Greaves D.R., Wang W., Dairaghi D.J., Dieu M.C., Saint-Vis B., Franz-Bacon K., Rossi D., Caux C., McClanahan T., Gordon S. (1997). CCR6, a CC chemokine receptor that interacts with macrophage inflammatory protein 3alpha and is highly expressed in human dendritic cells. J. Exp. Med..

[179] Liao F., Rabin R.L., Smith C.S., Sharma G., Nutman T.B., Farber J.M. (1999). CC-chemokine receptor 6 is expressed on diverse memory subsets of T cells and determines responsiveness to macrophage inflammatory protein 3 alpha. J. Immunol..

[180] Schwickert T.A., Victora G.D., Fooksman D.R., Kamphorst A.O., Mugnier M.R., Gitlin A.D., Dustin M.L., Nussenzweig M.C. (2011). A dynamic T cell-limited checkpoint regulates affinity-dependent B cell entry into the germinal center. J. Exp. Med..

[181] Dieu M.C., Vanbervliet B., Vicari A., Bridon J.M., Oldham E., Aït-Yahia S., Brière F., Zlotnik A., Lebecque S., Caux C. (1998). Selective recruitment of immature and mature dendritic cells by distinct chemokines expressed in different anatomic sites. J. Exp. Med..

[182] Sallusto F., Schaerli P., Loetscher P., Schaniel C., Lenig D., Mackay C.R., Qin S., Lanzavecchia A. (1998). Rapid and coordinated switch in chemokine receptor expression during dendritic cell maturation. Eur. J. Immunol..

[183] Caux C., Ait-Yahia S., Chemin K., de Bouteiller O., Dieu-Nosjean M.C., Homey B., Massacrier C., Vanbervliet B., Zlotnik A., Vicari A. (2000). Dendritic cell biology and regulation of dendritic cell trafficking by chemokines. Springer Semin. Immunopathol..

[184] Sulpice L., Desille M., Turlin B., Fautrel A., Boudjema K., Clément B., Coulouarn C. (2016). Gene expression profiling of the tumor microenvironment in human intrahepatic cholangiocarcinoma. Genom. Data.

[185] Oishi N., Kumar M.R., Roessler S., Ji J., Forgues M., Budhu A., Zhao X., Andersen J.B., Ye Q.H., Jia H.L. (2012). Transcriptomic profiling reveals hepatic stem-like gene signatures and interplay of miR-200c and epithelial-mesenchymal transition in intrahepatic cholangiocarcinoma. Hepatology.

[186] Bouma G., Zamuner S., Hicks K., Want A., Oliveira J., Choudhury A., Brett S., Robertson D., Felton L., Norris V. (2017). CCL20 neutralization by a monoclonal antibody in healthy subjects selectively inhibits recruitment of CCR6. Br. J. Clin. Pharmacol..

[187] Zhang W., Wang H., Sun M., Deng X., Wu X., Ma Y., Li M., Shuoa S.M., You Q., Miao L. (2020). CXCL5/CXCR2 axis in tumor microenvironment as potential diagnostic biomarker and therapeutic target. Cancer Commun..

[188] Cheng Y., Ma X.L., Wei Y.Q., Wei X.W. (2019). Potential roles and targeted therapy of the CXCLs/CXCR2 axis in cancer and inflammatory diseases. Biochim. Biophys. Acta Rev. Cancer.

[189] Okabe H., Beppu T., Ueda M., Hayashi H., Ishiko T., Masuda T., Otao R., Horlad H., Mima K., Miyake K. (2012). Identification of CXCL5/ENA-78 as a factor involved in the interaction between cholangiocarcinoma cells and cancer-associated fibroblasts. Int. J. Cancer.

[190] Imai T., Hieshima K., Haskell C., Baba M., Nagira M., Nishimura M., Kakizaki M., Takagi S., Nomiyama H., Schall T.J. (1997). Identification and molecular characterization of fractalkine receptor CX3CR1, which mediates both leukocyte migration and adhesion. Cell.

[191] Conroy M.J., Lysaght J. (2020). CX3CL1 Signaling in the Tumor Microenvironment. Adv. Exp. Med. Biol..

[192] Bazan J.F., Bacon K.B., Hardiman G., Wang W., Soo K., Rossi D., Greaves D.R., Zlotnik A., Schall T.J. (1997). A new class of membrane-bound chemokine with a CX3C motif. Nature.

[193] Garton K.J., Gough P.J., Blobel C.P., Murphy G., Greaves D.R., Dempsey P.J., Raines E.W. (2001). Tumor necrosis factor-alpha-converting enzyme (ADAM17) mediates the cleavage and shedding of fractalkine (CX3CL1). J. Biol. Chem..

[194] Hundhausen C., Misztela D., Berkhout T.A., Broadway N., Saftig P., Reiss K., Hartmann D., Fahrenholz F., Postina R., Matthews V. (2003). The disintegrin-like metalloproteinase ADAM10 is involved in constitutive cleavage of CX3CL1 (fractalkine) and regulates CX3CL1-mediated cell-cell adhesion. Blood.

[195] Meucci O., Fatatis A., Simen A.A., Miller R.J. (2000). Expression of CX3CR1 chemokine receptors on neurons and their role in neuronal survival. Proc. Natl. Acad. Sci. USA.

[196] Ferretti E., Bertolotto M., Deaglio S., Tripodo C., Ribatti D., Audrito V., Blengio F., Matis S., Zupo S., Rossi D. (2011). A novel role of the CX3CR1/CX3CL1 system in the cross-talk between chronic lymphocytic leukemia cells and tumor microenvironment. Leukemia.

[197] Imai T., Yasuda N. (2016). Therapeutic intervention of inflammatory/immune diseases by inhibition of the fractalkine (CX3CL1)-CX3CR1 pathway. Inflamm. Regen..

[198] Boonstra K., Weersma R.K., van Erpecum K.J., Rauws E.A., Spanier B.W., Poen A.C., van Nieuwkerk K.M., Drenth J.P., Witteman B.J., Tuynman H.A. (2013). Population-based epidemiology, malignancy risk, and outcome of primary sclerosing cholangitis. Hepatology.

[199] He X.S., Ansari A.A., Ridgway W.M., Coppel R.L., Gershwin M.E. (2006). New insights to the immunopathology and autoimmune responses in primary biliary cirrhosis. Cell Immunol..

[200] Krizhanovsky V., Yon M., Dickins R.A., Hearn S., Simon J., Miething C., Yee H., Zender L., Lowe S.W. (2008). Senescence of activated stellate cells limits liver fibrosis. Cell.

[201] Sasaki M., Miyakoshi M., Sato Y., Nakanuma Y. (2014). Chemokine-chemokine receptor CCL2-CCR2 and CX3CL1-CX3CR1 axis may play a role in the aggravated inflammation in primary biliary cirrhosis. Dig. Dis. Sci..

[202] Xie F., Feng S., Yang H., Mao Y. (2019). Extracellular vesicles in hepatocellular cancer and cholangiocarcinoma. Ann. Transl. Med..

[203] Raposo G., Stoorvogel W. (2013). Extracellular vesicles: Exosomes, microvesicles, and friends. J. Cell Biol..

[204] Yáñez-Mó M., Siljander P.R., Andreu Z., Zavec A.B., Borràs F.E., Buzas E.I., Buzas K., Casal E., Cappello F., Carvalho J. (2015). Biological properties of extracellular vesicles and their physiological functions. J. Extracell. Vesicles.

[205] Théry C., Ostrowski M., Segura E. (2009). Membrane vesicles as conveyors of immune responses. Nat. Rev. Immunol..

[206] Colombo M., Raposo G., Théry C. (2014). Biogenesis, secretion, and intercellular interactions of exosomes and other extracellular vesicles. Annu. Rev. Cell Dev. Biol..

[207] Kalluri R. (2016). The biology and function of exosomes in cancer. J. Clin. Investig..

[208] Willms E., Cabañas C., Mäger I., Wood M.J.A., Vader P. (2018). Extracellular Vesicle Heterogeneity: Subpopulations, Isolation Techniques, and Diverse Functions in Cancer Progression. Front. Immunol..

[209] Grange C., Tapparo M., Collino F., Vitillo L., Damasco C., Deregibus M.C., Tetta C., Bussolati B., Camussi G. (2011). Microvesicles released from human renal cancer stem cells stimulate angiogenesis and formation of lung premetastatic niche. Cancer Res..

[210] Mu W., Rana S., Zöller M. (2013). Host matrix modulation by tumor exosomes promotes motility and invasiveness. Neoplasia.

[211] Shao H., Chung J., Lee K., Balaj L., Min C., Carter B.S., Hochberg F.H., Breakefield X.O., Lee H., Weissleder R. (2015). Chip-based analysis of exosomal mRNA mediating drug resistance in glioblastoma. Nat. Commun..

[212] Chen W.X., Liu X.M., Lv M.M., Chen L., Zhao J.H., Zhong S.L., Ji M.H., Hu Q., Luo Z., Wu J.Z. (2014). Exosomes from drug-resistant breast cancer cells transmit chemoresistance by a horizontal transfer of microRNAs. PLoS ONE.

[213] Chalmin F., Ladoire S., Mignot G., Vincent J., Bruchard M., Remy-Martin J.P., Boireau W., Rouleau A., Simon B., Lanneau D. (2010). Membrane-associated Hsp72 from tumor-derived exosomes mediates STAT3-dependent immunosuppressive function of mouse and human myeloid-derived suppressor cells. J. Clin. Investig..

[214] Kogure T., Lin W.L., Yan I.K., Braconi C., Patel T. (2011). Intercellular nanovesicle-mediated microRNA transfer: A mechanism of environmental modulation of hepatocellular cancer cell growth. Hepatology.

[215] Baj-Krzyworzeka M., Szatanek R., Węglarczyk K., Baran J., Zembala M. (2007). Tumour-derived microvesicles modulate biological activity of human monocytes. Immunol. Lett..

[216] Baj-Krzyworzeka M., Weglarczyk K., Mytar B., Szatanek R., Baran J., Zembala M. (2011). Tumour-derived microvesicles contain interleukin-8 and modulate production of chemokines by human monocytes. Anticancer Res..

[217] Kahlert C., Melo S.A., Protopopov A., Tang J., Seth S., Koch M., Zhang J., Weitz J., Chin L., Futreal A. (2014). Identification of double-stranded genomic DNA spanning all chromosomes with mutated KRAS and p53 DNA in the serum exosomes of patients with pancreatic cancer. J. Biol. Chem..

[218] Dutta S., Reamtong O., Panvongsa W., Kitdumrongthum S., Janpipatkul K., Sangvanich P., Piyachaturawat P., Chairoungdua A. (2015). Proteomics profiling of cholangiocarcinoma exosomes: A potential role of oncogenic protein transferring in cancer progression. Biochim. Biophys. Acta.

[219] Hoshino A., Costa-Silva B., Shen T.L., Rodrigues G., Hashimoto A., Tesic Mark M., Molina H., Kohsaka S., Di Giannatale A., Ceder S. (2015). Tumour exosome integrins determine organotropic metastasis. Nature.

[220] Macias R.I.R., Banales J.M., Sangro B., Muntané J., Avila M.A., Lozano E., Perugorria M.J., Padillo F.J., Bujanda L., Marin J.J.G. (2018). The search for novel diagnostic and prognostic biomarkers in cholangiocarcinoma. Biochim. Biophys. Acta Mol. Basis Dis..

[221] Lapitz A., Arbelaiz A., Olaizola P., Aranburu A., Bujanda L., Perugorria M.J., Banales J.M. (2018). Extracellular Vesicles in Hepatobiliary Malignancies. Front. Immunol..

[222] Bian X., Xiao Y.T., Wu T., Yao M., Du L., Ren S., Wang J. (2019). Microvesicles and chemokines in tumor microenvironment: Mediators of intercellular communications in tumor progression. Mol. Cancer.

[223] Steinberg M., Silva M. (2010). Plerixafor: A chemokine receptor-4 antagonist for mobilization of hematopoietic stem cells for transplantation after high-dose chemotherapy for non-Hodgkin’s lymphoma or multiple myeloma. Clin. Ther..

[224] Schott A.F., Goldstein L.J., Cristofanilli M., Ruffini P.A., McCanna S., Reuben J.M., Perez R.P., Kato G., Wicha M. (2017). Phase Ib Pilot Study to Evaluate Reparixin in Combination with Weekly Paclitaxel in Patients with HER-2-Negative Metastatic Breast Cancer. Clin. Cancer Res..

[225] Yuan D., Huang S., Berger E., Liu L., Gross N., Heinzmann F., Ringelhan M., Connor T.O., Stadler M., Meister M. (2017). Kupffer Cell-Derived Tnf Triggers Cholangiocellular Tumorigenesis through JNK due to Chronic Mitochondrial Dysfunction and ROS. Cancer Cell.

